# N‐Doping Activated Presodiation Enhances Sodium‐Ion Provision in Hard Carbon Anodes

**DOI:** 10.1002/advs.75460

**Published:** 2026-06-22

**Authors:** Hua Lin, Wenxing Miao, Yanrong Shi, Guangyi Mao, Ding Ding, Jian Weng, Zhongxiong Fan, Qingchi Xu, Jun Xu

**Affiliations:** ^1^ School of Pharmaceutical Sciences Institute of Materia Medica Xinjiang University Urumqi China; ^2^ Department of Physics Research Institute for Biomimetics and Soft Matter Fujian Provincial Key Laboratory for Soft Functional Materials Xiamen University Xiamen China; ^3^ Shenzhen Research Institute of Xiamen University Shenzhen China; ^4^ The Higher Educational Key Laboratory for Biomedical Engineering of Fujian Province Research Center of Biomedical Engineering of Xiamen Department of Biomaterials College of Materials Xiamen University Xiamen China

**Keywords:** hard carbon, heteroatom doping, presodiation, sodium‐ion batteries, solid electrolyte interface

## Abstract

Hard carbon (HC) is considered the most promising anode material for commercial sodium‐ion batteries (SIBs), yet it still faces critical challenges such as low initial Coulombic efficiency (ICE). Presodiation can improve ICE by compensating for sodium loss caused by irreversible defects in HC. However, research on how the active sodium content during presodiation affects the formation and stability of the preconstructed solid electrolyte interface (SEI) remains limited. Based on this background, this work introduces many pyrrolic nitrogen (N‐5) sites into HC through pyrolysis of a urea‐HC composite, working in synergy with chemical presodiation to enhance sodium‐ion provision. Experimental and theoretical investigations reveal that the N‐5 sites strengthen the adsorption capability for Na^+^ and its ability to capture PF_6_^−^ from the electrolyte, promoting the preformation of a stable, inorganic‐rich SEI layer. As a result, the PS‐0.5NHC achieves an excellent ICE of 98.54%, a capacity retention of 90.6% after 1000 cycles at 1.5 A g^−1^. In a full‐cell configuration, the ICE is also significantly improved from 70.74% to 91.53%. This work provides a novel design concept for mitigating the inherent defects of the HC anode.

## Graphical abstract

Introducing pyrrolic‐N sites enables efficient presodiation and stronger PF_6_^−^ capture, thus pre ‐ forming a robust inorganic‐rich SEI layer in hard carbon anodes.

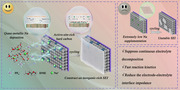

## 1 Introduction

The large‐scale iterative evolution of renewable energy systems and electrified transportation is driving explosive market demand for cost‐effective, sustainable energy storage technologies. Concurrently, price volatility stemming from the uneven geographical distribution of lithium resources and thermal runaway risks has prompted the academic community to shift its focus toward sodium‐ion batteries (SIBs) [1, 2]. Today, SIBs have emerged as a highly promising solution for grid‐scale energy storage and low‐cost applications due to their three core advantages: abundant resource reserves, low raw material costs, and outstanding safety performance coupled with excellent low‐temperature capabilities [3]. As a core component determining the energy density and cycle life of SIBs, the performance optimization of anode materials has consistently been a research focus [4]. Among these, HC stands out due to its unique microstructural advantages: its disordered carbon layer structure provides an optimal interlayer spacing (typically 0.37–0.40 nm), creating natural sites for the reversible insertion/de‐insertion of Na^+^ [5]. The abundant nanopores (including micropores and mesopores) can store additional Na^+^ through physical adsorption and chemical filling [6]. Based on a synergistic sodium storage mechanism involving adsorption, intercalation, and pore filling, HC exhibits high reversible sodium storage capacity and excellent cycling stability, making it the most promising SIBs anode material for industrialization to date [7, 8, 9, 10, 11, 12].

However, the practical application of HC remains constrained by three key scientific challenges: (1) Limited active sites (sites capable of reversible sodium storage) result in insufficient utilization of theoretical capacity [13]. (2) During the initial cycling process, surface defects on the HC undergo vigorous side reactions with the electrolyte (such as solvent molecule reduction and decomposition). Simultaneously, Na^+^ is irreversibly trapped within carbon layer defects or the newly formed solid electrolyte interphase (SEI). This results in an initial Coulombic efficiency (ICE) typically below 80%, severely limiting the energy density of the full‐cell [14, 15, 16]. (3) The SEI layer formed during the first cycle primarily consists of organic components such as alkyl carbonates. During prolonged charge–discharge cycles, it undergoes continuous restructuring due to volume expansion caused by repeated Na^+^ insertion/de‐insertion. This leads to a persistent increase in interfacial impedance, continuous depletion of active Na^+^, and rapid capacity decay [17, 18, 19, 20]. Simultaneously, it induces safety risks such as electrolyte gas evolution, localized overheating, and separator failure [21, 22]. A thorough analysis reveals that the root cause of these issues lies in the insufficient synergistic regulation between the structural characteristics of HC itself (distribution of active sites, defect types, and content) and the electrode‐electrolyte interface reactions (SEI layer formation mechanism, irreversible Na^+^ depletion pathways). A precise matching relationship between “structure‐interface‐performance” has yet to be established.

To address these critical issues, existing research strategies primarily focus on three key directions: “chemical modification,” “structural regulation,” and “interface optimization.” (1) By doping with non‐metallic elements (N, O, F), the electronic structure of HC is engineered to create reversible Na^+^ storage active sites, thereby enhancing capacity and conductivity [23, 24, 25, 26]. (2) Constructing closed nanoscale pores reduces dynamic contact between the electrolyte and the interior of HC, thereby stabilizing the SEI layer structure [27, 28, 29]. (3) Employ presodiation (chemical/electrochemical presodiation) to compensate for initial sodium loss and enhance ICE [30, 31, 32, 33]. However, these strategies often suffer from trade‐offs: while element doping can enhance capacity by creating active sites, the resulting defects exacerbate irreversible Na^+^ trapping, leading to a further decrease in ICE [34, 35, 36]. Although closed‐pore fabrication can reduce electrolyte side reactions, it inevitably extends the diffusion pathway of Na^+^, leading to degraded high‐rate performance [37]. Furthermore, single presodiation can only compensate for the initial Na^+^ depletion and cannot stabilize the SEI layer. To preconstrust a stable SEI structure enriched with inorganic compounds (such as NaF), additional film‐forming additives are typically required [38]. This has increased battery production costs to some extent. Therefore, to address the research gap in the synergistic regulation of “structure‐interface‐performance,” developing a multifunctional strategy that simultaneously optimizes the intrinsic structure of HC and regulates the electrode‐electrolyte interface has become an urgent priority [39]. Previous studies indicate that the sodium element in the SEI layer primarily originates from the dissociation of Na salts in the electrolyte and the release of Na^+^ during electrode redox reactions in charging and discharging cycles [30, 40, 41]. Since the former requires overcoming solvation energy to escape the solvation shell, it struggles to participate in the preconstrusted SEI [20, 42].

**FIGURE 1:**
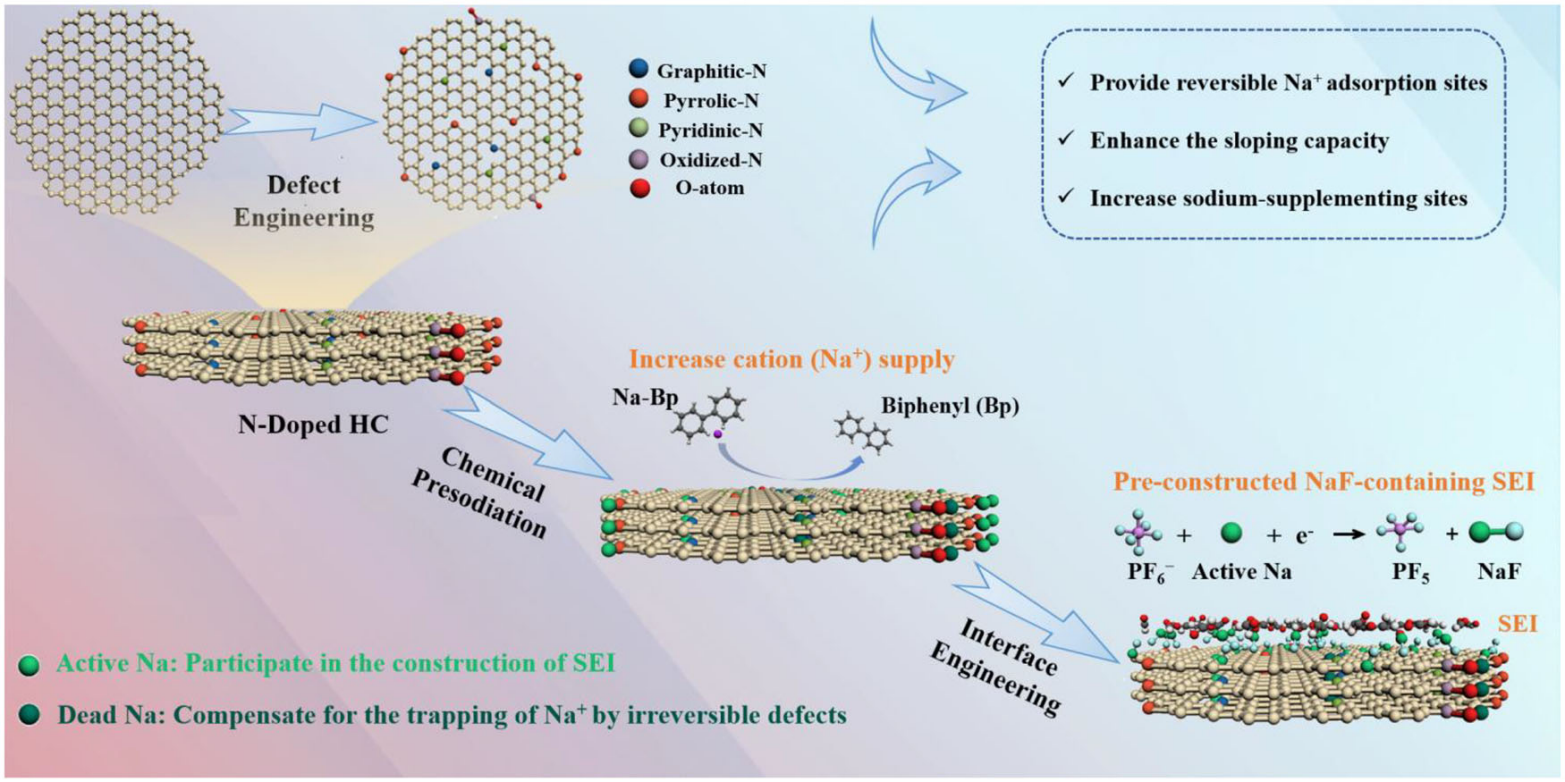
Schematic illustration of the core strategy for synergistic regulation of hard carbon anode by N‐Doping and presodiation.

In this work, a synergistic regulation strategy centered on enhancing cation supply is proposed to address issues such as poor cycling stability and low initial coulombic efficiency (ICE) in sodium‐ion batteries (SIBs) with hard carbon (HC) anodes (Figure 1). Pyrolytic introduction of pyrrolic nitrogen (N‐5) active sites was achieved through a urea/HC composite, while chemical presodiation was concurrently employed to increase sodium replenishment content. Experimental and theoretical investigations revealed that this approach enhances the reversible adsorption/desorption of Na^+^ on HC, simultaneously strengthens the adsorption capacity for PF_6_^−^ anions from the electrolyte during presodiation, and promotes the preformation of a stable inorganic‐rich solid electrolyte interphase (SEI). These synergistic effects not only increase the sodium storage capacity but also improve the kinetic properties of the electrode‐electrolyte interface. Thanks to these advantages, the PS‐0.5NHC demonstrated a high ICE of 98.54% at 0.03 A g^−1^, and a capacity retention of 90.6% after 1000 cycles at 1.5 A g^−1^. Furthermore, the ICE of the full‐cell increased significantly from 70.74% to 91.53%.

## 2 Results and Discussion

As shown in Figure S1, the synthesis process of a series of N‐doped HC and the specific operations for presodiation are illustrated. Figure S3 shows the SEM images of a series of HC anodes. Advanced N‐doped HC (denoted as “XNHC,” where “X” represents the mass ratio of urea to the HC precursor: 0.2NHC, 0.5NHC, 0.8NHC) anodes were prepared based on the principle of urea‐assisted pyrolysis, wherein urea serves both as a nitrogen dopant and a chemical activator. During high‐temperature treatment, the decomposition of urea releases reactive species (e.g., NH_3_, HCN) that facilitate the in situ incorporation of N into the HC framework. This process is meticulously optimized to generate a specific defect architecture dominated by pyrrolic nitrogen (N‐5) configurations, which concurrently induces lattice distortion to expand the interlayer spacing and creates abundant reversible adsorption sites. The presodiation operation mechanism leverages these engineered sites through a solution‐based chemical reduction approach using sodium biphenyl (Na‐Bp). The strongly reducing biphenyl radical anion (Bp^·−^) spontaneously donates electrons to the HC anode, accompanied by the adsorption and stabilization of Na^+^ primarily at the N‐5 defects.

**FIGURE 2:**
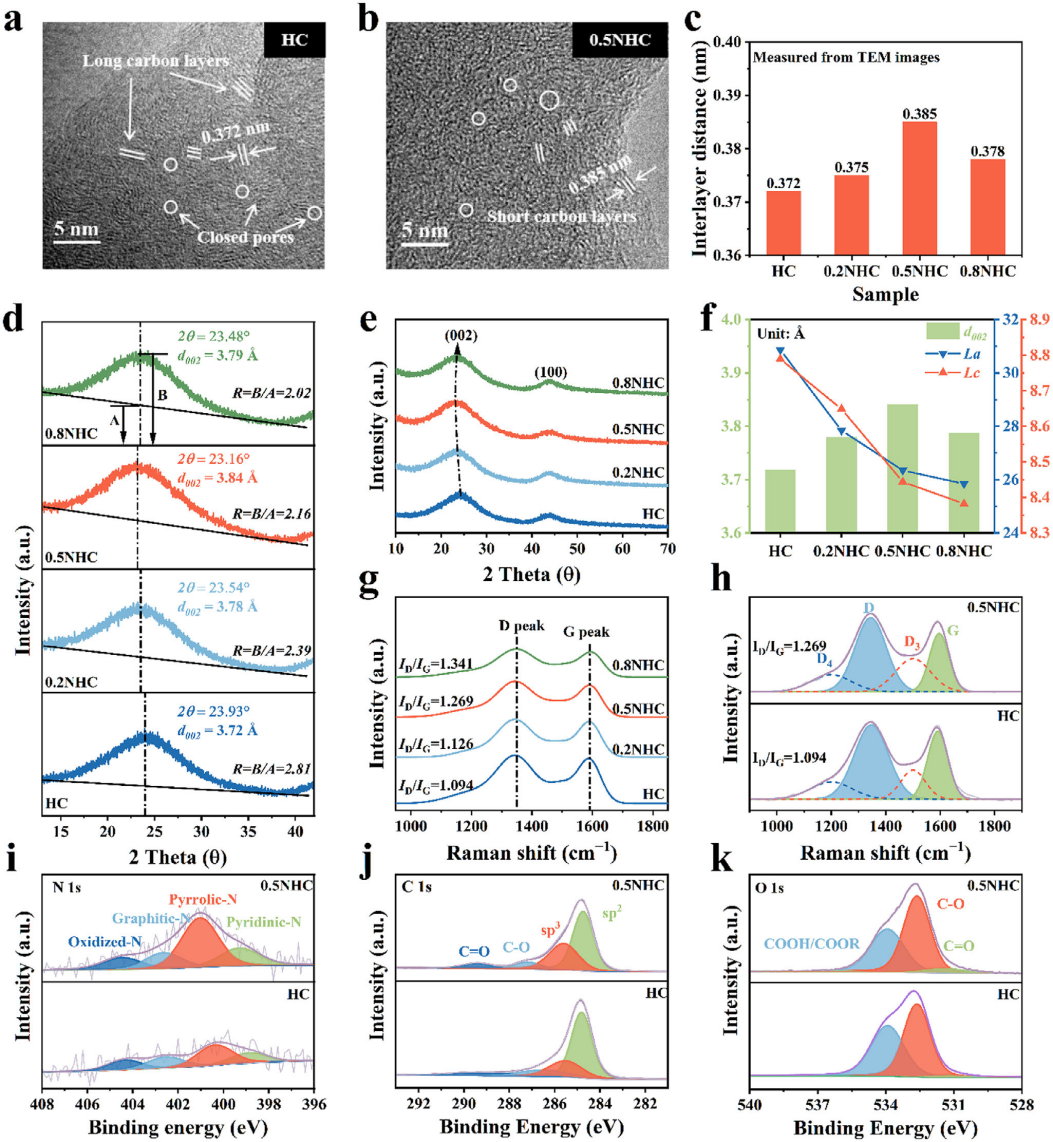
HRTEM images of (a) HC and (b) 0.5NHC. (c) The interlayer distances were measured from TEM images of HC and XNHC. (d) R values and (e) XRD spectra of HC and XNHC. (f) L_a_, L_c_, and d_002_ values calculated from XRD. (g) Raman spectra of HC and XNHC. (h) Raman Spectra with peak fitting of HC and 0.5NHC. (i) High‐resolution N 1s XPS spectra of HC and 0.5NHC. (j) Corresponding C 1s spectra. (k) Corresponding O 1s spectra.

The microstructure of XNHC and HC was characterized using a high‐resolution transmission electron microscope (HRTEM). As shown in Figure 2a,b, the HC sample without urea addition exhibits extensive long‐range ordered vortex‐like graphitic domain structures and abundant pores (Figure S4). The longer graphitic domain carbon layers indicate that the HC formed at 1200°C possesses highly graphitized and ordered characteristics (the rationale for selecting 1200°C is provided in Figure S2). As the percentage of urea increases, the order of the N‐doped HC material gradually decreases, and the graphitic domains become progressively shorter. These short‐range graphitic domains stack in a layered arrangement, forming numerous closed pores. Additionally, interlayer spacing measurements obtained via high‐resolution transmission electron microscopy reveal (Figure 2c): The carbon interlayer spacings for HC, 0.2NHC, 0.5NHC, and 0.8NHC are 0.372, 0.375, 0.385, and 0.378 nm, respectively. This may be attributed to nitrogen atoms in N‐doped HC providing additional electrons to the carbon π system, thereby increasing the interlayer electron density. This, in turn, enhances the electrostatic repulsion between layers, leading to an expanded interlayer spacing [43]. At a mass ratio of urea to coconut shell charcoal of 0.5, the maximum interlayer spacing reached 0.385 nm. However, when the mass ratio increased to 0.8, the excess urea converted a significant amount of amorphous carbon into volatile products, causing the carbon framework to collapse. Under the influence of van der Waals forces, graphite‐like layers slipped and densely stacked, thereby reducing the interlayer spacing [44]. Scanning electron microscopy (SEM) and energy dispersive spectroscopy (EDS) images of XNHC and HC (Figure S5) confirm the presence of N elements.

X‐ray diffraction (XRD) patterns reveal broadened peaks for both XNHC and HC at ∼23° and ∼43°, corresponding to the (002) and (100) lattice planes of graphite nanodomains—a characteristic feature of disordered carbon materials (Figure 2d,e). Notably, the (002) peak exhibits an overall shift as the urea content increases. According to the Bragg law [45], the following values were calculated: The interlayer spacings for HC, 0.2NHC, 0.5NHC, and 0.8NHC were determined to be 0.372, 0.378, 0.384, and 0.379 nm, respectively. These results align with the interlayer spacing trends observed via HRTEM. Larger interlayer spacings provide more Na^+^ storage sites and reduce the transfer resistance [46]. In addition to variations in interlayer spacing, we also compared graphitization levels through changes in the R value [47]. The higher the R value, the greater the degree of graphitization and order. Figure 2f demonstrates that the 0.5NHC material exhibits the most suitable L_a_, L_c_, and d_002_ values, confirming that urea content plays a crucial role in the structure of N‐doped HC. Furthermore, we evaluated the disorder and defect states of these four materials based on Raman spectroscopy. As shown in Figure 2g,h and Figure S6, the Raman spectrum was deconvoluted into four peaks. The D_3_ peak (∼1500 cm^−1^) originates from vibrations directly related to the interlayer stacking mode of multilayer graphene (or graphite‐like carbon layers). The D_4_ peak (∼1200 cm^−1^) originates from sp^2^ carbon π‐plane symmetry breaking signals induced by heteroatom incorporation. The D peak (∼1350 cm^−1^) correlates with sp^3^ hybridization defects and disorder, while the G peak (∼1580 cm^−1^) corresponds to in‐plane stretching vibrations of sp^2^‐hybridized carbon atoms in the carbon material. The I_D_/I_G_ ratio (the ratio of D peak to G peak intensity) serves as a key parameter characterizing material disorder and graphitization degree. A higher ratio indicates a greater defect density within the material [48]. The I_D_/I_G_ ratio of the undoped HC sample is 1.094, indicating that the pristine HC skeleton primarily consists of low‐defect sp^2^ carbon. As the proportion of urea (nitrogen source) increases, the I_D_/I_G_ ratios for 0.2NHC, 0.5NHC, and 0.8NHC gradually rise to 1.126, 1.269, and 1.341, respectively. This occurs because nitrogen atoms replace sp^2^ carbon atoms in the HC skeleton, forming doping sites such as pyrrolic nitrogen (N‐5) and pyridine nitrogen (N‐6) (subsequently confirmed by XPS). This disrupts the periodic arrangement of the hexatomic rings, introducing a large number of sp^3^ defects, ultimately leading to a significant enhancement in the intensity of the D peak.

To further investigate the structural changes in nitrogen‐doped carbon materials, XPS analysis was employed to examine the N doping configurations and defect densities in XNHC and HC. The survey spectra reveal that the total nitrogen content initially increases with urea addition, reaching the highest value in 0.5NHC (2.22 at.%), but decreases significantly in 0.8NHC (1.02 at.%) due to excessive gasification of urea (e.g., NH_3_, HCN) and structural etching during pyrolysis (Figure S7). The high‐resolution N 1s spectra of the four materials reveal four configurable forms: pyridinic‐N (N‐6, 398.7 eV), pyrrolic‐N (N‐5, 400.4 eV), graphitic‐N (C─N, 402.3 eV), and oxidized‐N (N─O, 404.2 eV) (Figure 2i). At 1200°C, almost no N signal was detected in the HC sample without urea addition, whereas the nitrogen in 0.5NHC primarily exists as N‐5. The five‐membered ring structure of N‐5 generates a local strain field, inducing carbon lattice distortion and leading to an interlayer expansion effect. This mechanism contributes to the increased interlayer spacing observed after nitrogen doping [49, 50]. However, for 0.8NHC, excessive urea causes severe etching of the carbon framework, resulting in reduced nitrogen doping efficiency and a lower absolute N‐5 content. Despite its higher overall defect density, this ultimately limits the availability of reversible sodium storage sites in 0.8NHC. In the C 1s fine spectrum (Figure 2j), four components can be convoluted: sp^2^‐hybridized graphitized carbon (∼284.7 eV), sp^3^‐hybridized defective carbon (∼285.4 eV), C─O bonds (∼286.8 eV), and C═O bonds (∼289.5 eV) (Figure S8b). As the urea content increases, the sp^3^/sp^2^ ratio gradually rises (Table S1), confirming that N atoms induce distortion of the graphitic structure during carbonization with coconut shell charcoal, leading to the formation of abundant sp^3^‐hybridized defective carbon. As shown in Figure 2k, the O1s spectrum reveals contributions from C═O (∼531.5 eV) and C─O (∼532.6 eV) functional groups (Figure S8c). The presence of urea promotes the C═O functional group during high‐temperature carbonization by facilitating the conversion of C─O bonds to C═O bonds. This occurs because the N‐5 reduces the electron density on adjacent carbon atoms, making them more readily bondable with oxygen to form double bonds (C═O). We also conducted N_2_ adsorption‐desorption tests on four samples. As shown in Figure S9, the adsorption–desorption isotherms for HC, 0.2NHC, and 0.5NHC exhibit similar overall shapes with slightly increased adsorption capacities (Figure S9a–c). This may be attributed to increased defects creating more N_2_ adsorption sites. In contrast, the adsorption–desorption isotherm for the 0.8NHC sample (Figure S9d) exhibited a distinct non‐closure phenomenon. This likely stems from excessive urea increasing the defect density of the material, leading to strong interactions with N_2_. Consequently, N_2_ failed to desorb completely during the desorption process. The specific surface areas of four samples—HC, 0.2NHC, 0.5NHC, and 0.8NHC—were calculated using the Brunauer–Emmett–Teller (BET) method, yielding values of 3.035, 5.110, 6.097, and 36.511 m^2^ g^−1^, respectively. The increase in specific surface area stems from the progressive decomposition of urea during high‐temperature pyrolysis, yielding reactive species such as NH_3_, HCN, and CO_2_. Some nitrogen atoms from NH_3_ incorporate into the carbon framework via substitution reactions (forming N‐5, etc.), while the remaining reactive species react with the carbon framework, etching the carbon layers and promoting mesopore formation [44]. Therefore, in the pore size distribution (Figure S10), the mesoporous volume increases with the rising urea content, ultimately forming a multi‐level pore structure where micropores and mesopores coexist, thereby enhancing the specific surface area of the HC. However, when the mass ratio of urea to coconut shell carbon reaches 0.8, excessive urea generates active substances that excessively etch the carbon layers, fracturing them into “carbon nanofragments.” The resulting accumulation of “stacked mesopores” between these fragments causes a sudden surge in specific surface area. This promotes side reactions on the surface of the HC, which is detrimental to reversible sodium storage.

Based on the structural characterization, it can be concluded that the urea‐assisted pyrolyzed nitrogen‐doped HC exhibits significant changes in carbon framework structure and defect density compared to the initial HC. On one hand, N doping expands the interlayer spacing in graphitic domains of HC, providing storage sites and diffusion pathways for sodium ions. On the other hand, nitrogen atoms in the doping primarily exist as N‐5, simultaneously generating abundant sp^3^‐hybridized defective carbon. These defects dominated by N‐5 can serve as sodium adsorption centers during the presodiation process. Among the XNHC series samples, the 0.5NHC sample exhibits the most suitable comprehensive structural characteristics for sodium ion storage: Its maximum carbon layer spacing (0.384 nm) synergizes with a multi‐level pore structure to reduce Na^+^ diffusion resistance while providing ample insertion space. Combined with a moderate specific surface area and defect density, this configuration establishes highly efficient sodium storage sites, offering additional sodium‐replenishment sites for subsequent presodiation processes.

**FIGURE 3:**
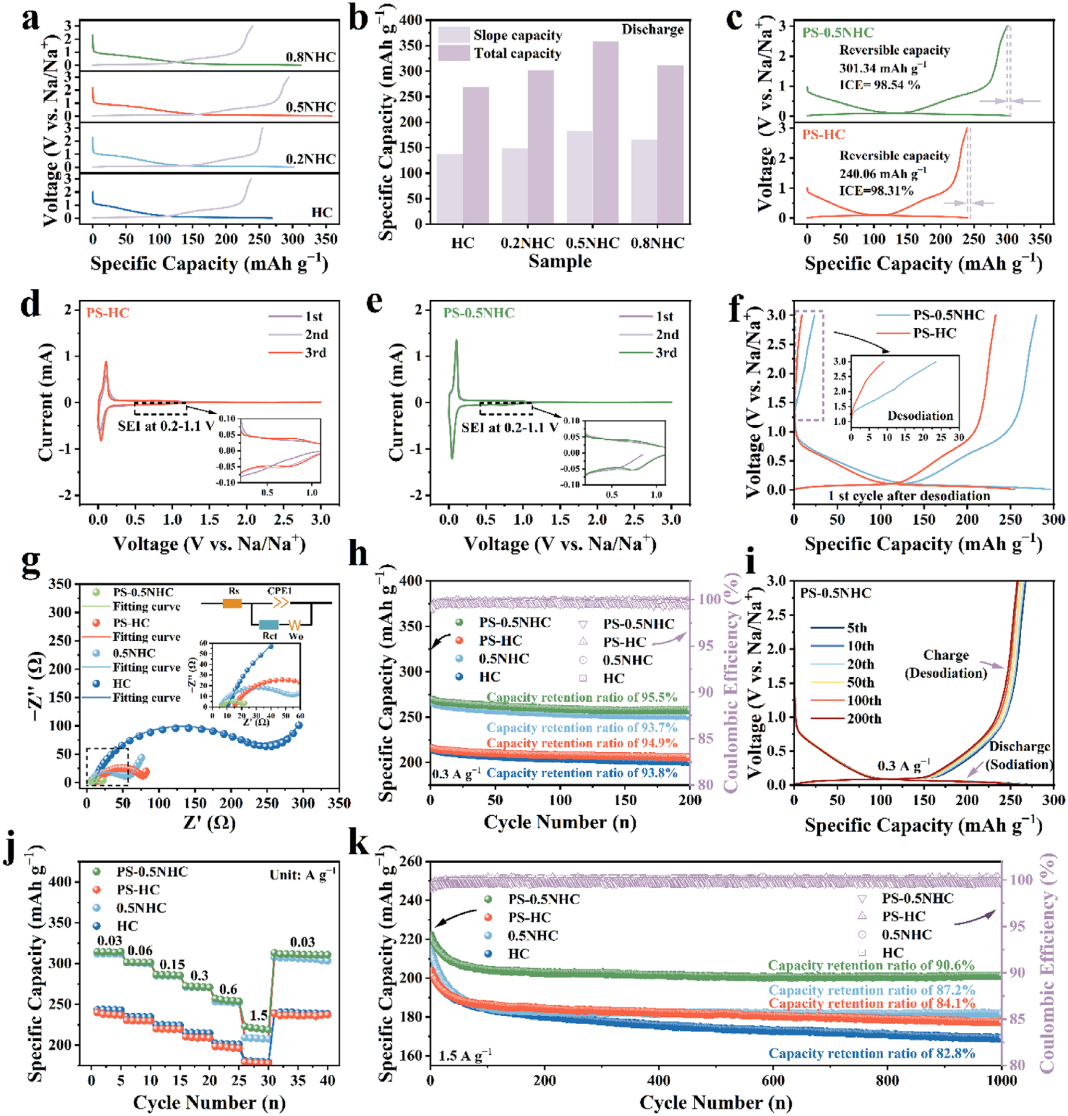
(a) Initial GCD curves and (b) corresponding slope discharge capacity (voltage > 0.1 V) of HC and XNHC. (c) Initial GCD, (d,e) CV, and (f) desodiation test curves of PS‐HC and PS‐0.5NHC. (g) EIS and (h) cycling performance of HC, 0.5NHC, PS‐HC, and PS‐0.5NHC. (i) GCD curves of PS‐0.5NHC. (j) Rate performance of HC, 0.5NHC, PS‐HC, and PS‐0.5NHC. (k) long‐cycling performance of HC, 0.5NHC, PS‐HC and PS‐0.5NHC at 1.5 A g^−1^.

From the initial galvanostatic charge–discharge (GCD) curves (Figure 3a), it is evident that the undoped HC exhibits a discharge specific capacity of only 268.49 mAh g^−1^ at 0.03 A g^−1^, with a reversible capacity further reduced to 237.37 mAh g^−1^ and an ICE of 88.4% (Figure S11). Among the series of N‐doped HC materials, the 0.5NHC exhibits structural and performance characteristics highly compatible with presodiation requirements: On one hand, the expanded carbon interlayer spacing resulting from nitrogen doping provides ample intercalation space for the early insertion of Na^+^ during the presodiation process while preserving the intact carbon framework structure, thereby preventing structural collapse caused by excessive defects; On the other hand, the N‐5‐dominated active sites introduced by nitrogen doping serve not only as conventional core active centers for sodium storage but also as dedicated active sodium source sites for presodiation. These sites stabilize the Na^+^ provided in the presodiation solution through strong adsorption (Figure S12). Consequently, the 0.5NHC anode exhibits a high discharge specific capacity of 358.28 mAh g^−1^, with the reversible capacity enhanced to 294.23 mAh g^−1^. Based on systematic structure‐property correlation analysis, 0.5NHC was selected as the optimal precursor for presodiation (Figure S13). Although doping introduces some irreversible defects that capture Na^+^, reducing the ICE to 82.1%, this loss can be compensated for by the subsequent presodiation process. Furthermore, this is further validated from the perspective of slope capacity contribution (Figure 3b): The slope capacity of the 0.5NHC anode during discharge (voltage > 0.1 V) reached 181.98 mAh g^−1^, significantly higher than that of HC (137.15 mAh g^−1^), 0.2NHC (148.10 mAh g^−1^), and 0.8NHC (165.18 mAh g^−1^). This portion of ramp capacity primarily originates from surface defect adsorption (at edges and defect sites) and interlayer insertion [7, 51]. These two sodium storage modes align perfectly with the presodiation mechanism for sodium source replenishment. During presodiation, the additional Na^+^ introduced can rapidly adsorb onto the active sites of 0.5NHC via surface adsorption. Subsequently, they are embedded within the carbon layer and stably retained within the carbon framework, forming a “reserve sodium source.” [52]

We use Na‐Bp as the chemical presodiation agent. Sodium metal (Na) and biphenyl (Bp) were dissolved in ethylene glycol dimethyl ether (DME) at a concentration of 0.5 mol L^−1^ to prepare the Na‐Bp‐DME solution [31]. As shown in Figure S14a, the solution exhibited a deep blue color. This is because Bp possesses strong electron affinity, while metallic sodium, as a potent reducing agent, spontaneously donates electrons to it, forming the biphenyl radical anion (Bp^·−^). Consequently, the solution exhibits a blue color. The observation of the Landé factor g = 2.003 via electron paramagnetic resonance (EPR) spectroscopy, characteristic of typical organic radical signals (Figure S14b), confirms the formation of the biphenyl radical anion. The reduction potential of the Bp/Bp^−^ redox pair in the Na‐Bp‐DME chemical presodiation solution was measured to be 0.301 V (Figure S14c) using cyclic voltammetry, which is significantly lower than the open‐circuit voltage of the anode half‐cell (∼2.2 V). thus enabling spontaneous transfer of Na^+^ and electrons to the anode material driven by the potential difference, thereby achieving presodiation.

We can control the degree of presodiation by adjusting the immersion time of the negative electrode plate (Figure S15). The degree of presodiation can be determined by the ICE of the PS‐0.5NHC//Na half‐cell. Additionally, a correlation exists between the degree of presodiation and the open‐circuit voltage of the PS‐0.5NHC//Na half‐cell (Figure S16a). The higher the degree of presodiation (i.e., the greater the sodium supplementation), the more significantly the ICE of the PS‐0.5NHC//Na half‐cell increases, while its open‐circuit voltage (OCV) gradually decreases. When the presodiated electrode is immersed in an ethanol solution containing phenolphthalein, the solution gradually turns red (Figure S16b), with the red color deepening as the immersion time increases. Ultimately, we selected 30 s as the optimal presodiation time to achieve an ICE approaching 100% in the PS‐0.5NHC//Na half‐cell (reached 98.54%). When ICE exceeds 100%, excessive Na^+^ insertion may cause localized Na^+^ concentration saturation at the electrode surface, triggering metallic Na deposition and resulting in dendrite formation—posing potential safety hazards during battery operation [53]. Similarly, presodiation the HC anode sheet also achieved an initial efficiency close to 100% (reached 98.31%). The charge–discharge curves for both electrodes are shown in Figure 3c. When the ICE approached 100%, the reversible capacity of the PS‐0.5NHC anode reached 301.34 mAh g^−1^, while that of the PS‐HC anode was only 240.06 mAh g^−1^. The slight increase in reversible capacity after presodiation for both electrodes is attributed to interbatch variations in the preparation process and does not affect the conclusions. We employed Raman spectroscopy, cyclic voltammetry (CV) testing, and electrochemical desalination testing to evaluate the sodium replenishment behavior during the presodiation process. Raman spectroscopy revealed the occupation of defect sites by sodium ions through the intensity ratio of the D and G peaks. Following presodiation, the I_D_/I_G_ ratio of PS‐0.5NHC decreased from 1.269 in 0.5NHC to 1.078, while that of PS‐HC decreased only from 1.094 to 0.999 (Figure S17), indicating that defect sites dominated by N‐5 in 0.5NHC are more readily occupied by replenished Na^+^. CV tests were conducted at a scan rate of 0.1 mV s^−1^ (voltage range: 0.01–3 V), as shown in Figure 3d,e, depicting the first three cycles of CV curves for HC, 0.5NHC, PS‐HC, and PS‐0.5NHC (Figure S18). During the first cycle, both the CV curves of HC and 0.5NHC exhibited a distinct irreversible reduction peak near 0.7 V, attributed to irreversible adsorption reactions between Na^+^ and irreversible defect sites on the HC surface, as well as the formation of the SEI. The CV curves of presodiated PS‐HC and PS‐0.5NHC exhibit the disappearance of irreversible reduction peaks at 0.7 V, indicating that the sodium source supplemented during presodiation occupies these irreversible defect sites and may have pre‐formed an SEI structure upon electrolyte contact. To investigate the reversible adsorption characteristics of Na^+^ supplemented by presodiation, we conducted desodiation tests. The presodiated PS‐HC and PS‐0.5NHC anodes were subjected to an initial GCD test, first charged to 3 V to facilitate the release of Na^+^ supplemented during presodiation. As shown in Figure 3f, both anodes can release sodium ions during the initial desorption process, demonstrating that the supplemented Na sources in both materials contain reversibly adsorbed components (referred to as reversible Na sources). Notably, the sodium desorption capacity of PS‐0.5NHC reaches 23.61 mAh g^−1^, significantly higher than that of PS‐HC at 9.06 mAh g^−1^. We utilized XPS to test the sodium content in the presodiated electrode sheets (prior to electrolyte contact). XPS analysis revealed that the signal intensity of the Na 1s characteristic peak (∼1072 eV) in the PS‐0.5NHC anode was significantly higher than that in PS‐HC (Figure S19). Its sodium content (7.4%) increased by 48.6% compared to PS‐HC (4.98%) (Figure S20). This result directly confirms that N‐doped active sites (such as pyrrole N defects) can efficiently adsorb and reduce Na^+^ during the presodiation process, thereby increasing the supply of sodium sources. Therefore, during the presodiation process (when the electrode sheet has not yet contacted the electrolyte), the supplemented Na^+^ simultaneously occupy both irreversible defect sites and reversible active sites. After the electrode sheet contacts the electrolyte, the Na^+^ at the reversible active sites participate in the preformation of the SEI layer. Meanwhile, the PS‐0.5NHC, possessing a more abundant active sodium source, significantly enhances the Na^+^ supply density, thereby enabling more substantial participation in the SEI layer formation process. Electrochemical testing was conducted on HC, 0.5NHC, PS‐HC, and PS‐0.5NHC anodes to analyze the synergistic regulation effect of N‐doping and presodiation. In electrochemical impedance spectroscopy (Figure 3g), the high‐frequency intercept reflects Ohmic resistance (*R*_s_) of the electrolyte and cell components, with all samples showing similar *R*_s_, indicating presodiation and N‐doping barely affect electrolyte resistance. The semicircle diameter corresponds to charge transfer resistance (*R*_ct_): unpresodiated HC has the largest *R*_ct_, while 0.5NHC reduces it slightly. After presodiation, PS‐HC and PS‐0.5NHC show much smaller *R*_ct_, with PS‐0.5NHC the smallest. The reduction in *R*_ct_ after presodiation is attributed to the formation of a uniform and stable SEI layer on the electrode surface, which facilitates charge transfer at the interface. Nitrogen doping further enhances this effect by introducing additional active sites and improving the electronic conductivity of the carbon framework, leading to a synergistic optimization of the electrode‐electrolyte interface. Presodiation occupies irreversible defect sites through sodium supplementation and pre‐builds the SEI layer to reduce side reactions at the interface. PS‐0.5NHC, owing to its abundant active Na source, participates in prebuilding the SEI while retaining sufficient Na^+^ to mitigate “sodium source deficiency” during charge transfer processes. EIS testing confirms that nitrogen doping (structural modification) and presodiation (interface optimization) exhibit a synergistic effect where their combined contribution to regulating the impedance of HC anodes yields a synergistic effect where the whole is greater than the sum of its parts. The detailed fitting parameters are provided in Table S2, with chi‐squared (χ^2^) values on the order of 10^−4^, confirming the reliability of the fitting results. Figure 3h,i and Figure S21a–c compare the cycling performance of four electrodes at 0.3 A g^−1^. The PS‐0.5NHC exhibits the best cycling stability and the highest reversible capacity. After 200 cycles, the capacity ranking was PS‐0.5NHC (257.88 mAh g^−1^) > 0.5NHC (251.63 mAh g^−1^) > PS‐HC (205.83 mAh g^−1^) > HC (200.44 mAh g^−1^). Moreover, the PS‐0.5NHC electrode maintained a high capacity retention rate of 95.5%. The rate performance of electrodes was demonstrated through five charge–discharge cycles at varying current densities (0.03 A g^−1^ to 1.5 A g^−1^). As shown in Figure 3j N‐doping enhances the content of N‐5 and active adsorption sites in HC at 0.03 A g^−1^, thereby promoting charge transfer and ion diffusion rates. This results in comparable specific capacities for 0.5NHC and PS‐0.5NHC, reaching 312.01 mAh g^−1^ and 314.61 mAh g^−1^, respectively. In contrast, HC and PS‐HC exhibited capacities of only 243.28 and 240.06 mAh g^−1^. When the current density reaches 1.5 A g^−1^, due to the stability of the preconstructed SEI, PS‐0.5NHC exhibits higher capacity retention than 0.5NHC. Specifically, PS‐0.5NHC has a capacity of 222.97 mAh g^−1^, while that of 0.5NHC is only 209.18 mAh g^−1^. When the current density was reduced back to 0.03 A g^−1^, PS‐0.5NHC also maintained excellent reversible capacity. Similarly, compared to the other two materials, PS‐0.5NHC exhibits superior rate performance. Notably, the rate capability of PS‐0.5NHC surpasses most previously reported coconut‐shell‐derived hard carbon anodes, particularly at high current densities (Figure S22 and Table S3), further confirming the effectiveness of our synergistic N‐doping and presodiation strategy. In addition, after being activated at a small current density of 0.03 A g^−1^ for three cycles, PS‐0.5NHC exhibited a reversible capacity of 201.37 mAh g^−1^ with a high capacity retention rate of 90.6% after 1000 cycles at 1.5 A g^−1^, demonstrating outstanding long‐term cycling performance (Figure 3k). In contrast, the capacity retention rates for HC, 0.5NHC, and PS‐HC were only 82.8%, 87.2%, and 84.1%, respectively.

**FIGURE 4:**
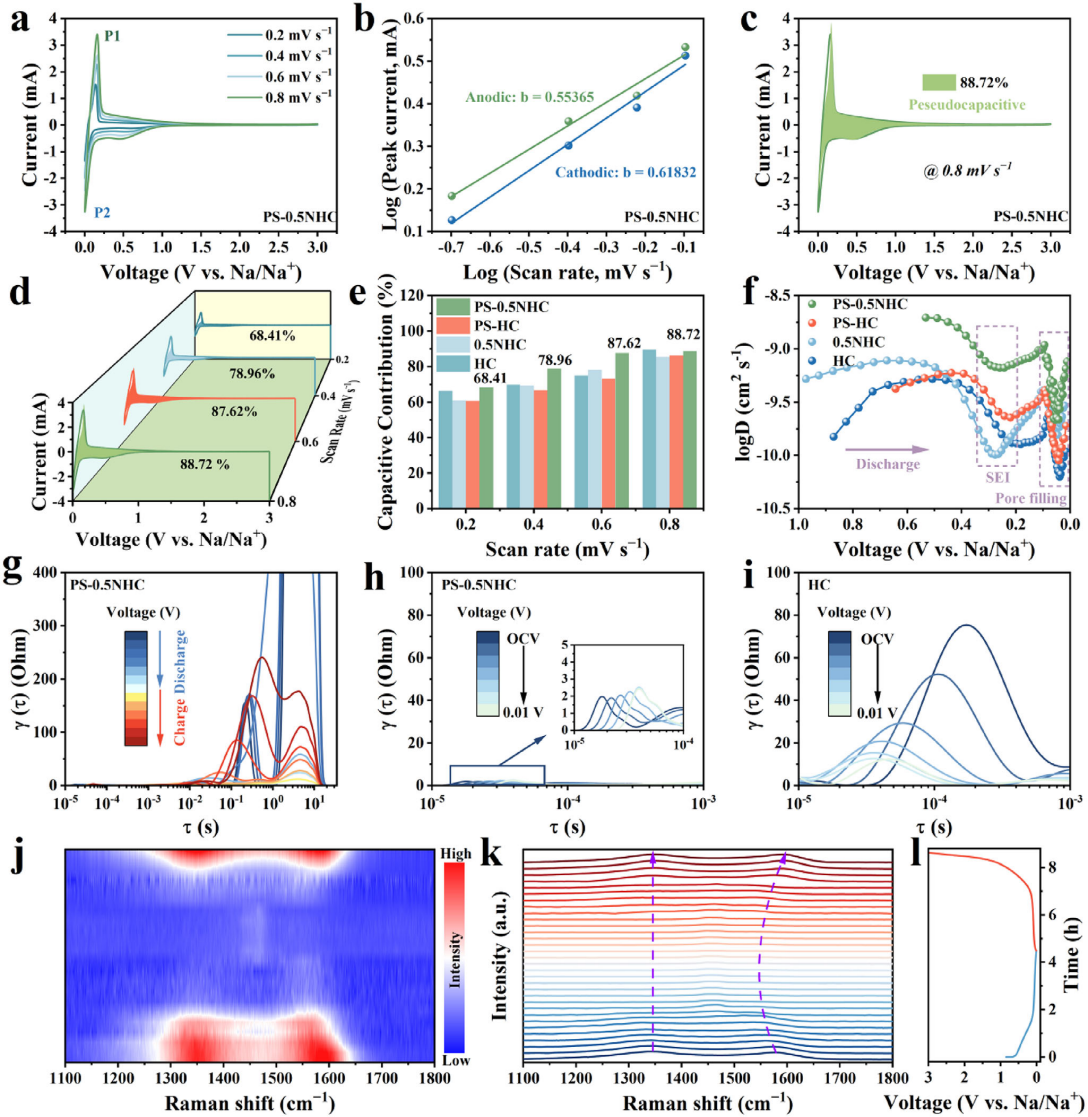
(a) CV curves and (b) the corresponding linearity relationship between log(i) and log(ν) for the cathodic and anodic peaks of PS‐0.5NHC anode. (c,d) Capacitive contribution at various scan rates for PS‐0.5NHC anodes. (e) The contribution ratios of capacitive capacity at various scan rates and (f) sodium ion diffusion coefficient (D_Na+_) of HC, 0.5NHC, PS‐HC, and PS‐0.5NHC anodes. (g–i) Relaxation time distribution (DRT) curves of PS‐0.5NHC and HC anodes. (j,k) In situ Raman spectra of PS‐0.5NHC anode synchronized with (l) GCD curve.

To further elucidate the sodium storage kinetic advantages of the PS‐0.5NHC, cyclic voltammetry (CV) testing and galvanostatic intermittent titration technique (GITT) were conducted on HC, 0.5NHC, PS‐HC, and PS‐0.5NHC at different scan rates. This revealed the kinetic regulation mechanisms from both the “interfacial reaction mechanism” and “bulk ion diffusion” dimensions. In the CV curves recorded at different scan rates (Figure S23), as the scan rate increased from 0.2 to 0.8 mV s^−1^, the redox peaks (sodium deintercalation peak P_1_ at ∼0.142 V and sodium intercalation peak P_2_ at ∼0.01 V) of the PS‐0.5NHC exhibited significantly increased currents, while maintaining good peak symmetry, indicating stable interfacial reactions (Figure 4a). The peak current (i) of the electrode reaction followed a power‐law relationship with the scan rate (v), as shown in Equation (1) [54]. In the equation, ɑ is a constant related to the electrode diffusion coefficient and active area; the exponent b is a key parameter for determining the control mechanism of the electrode reaction (diffusion‐controlled or surface‐controlled).

(1) $$i=av^{b}$$

The value of b can be determined by fitting the slope of the log(i)‐log(v) plot. When b = 1, the process is capacitive and surface adsorption‐controlled; when b = 0.5, it is diffusion‐controlled and insertion‐type. Fitting calculations yielded b values of 0.62 and 0.55 for the anode peak (0.142 V) and cathode peak (0.01 V) of PS‐0.5NHC, respectively (Figure 4b). This indicates that during the low‐voltage plateau, the sodium storage process is predominantly diffusion‐controlled, accompanied by a capacitive component. Other samples (HC, 0.5NHC, PS‐HC) also exhibit “diffusion‐capacitive synergistic control” characteristics, as shown in Figure S24. However, the precise contribution ratios of the two electrochemical processes throughout the charge–discharge cycle can be calculated using Equation (2) [55].

(2) $$i=k_{1}v+k_{2}v^{1/2}$$

k_1_v corresponds to the current contribution from the capacitive control process, while k_2_v^1/2^ corresponds to the current contribution from the diffusion‐controlled process. As the scan rate increases, the capacitive contribution of the PS‐0.5NHC electrode continues to rise (Figure 4d), indicating that the interfacial capacitive process becomes dominant at high scan rates, with reaction kinetics playing a more prominent role. At a scan rate of 0.8 mV s^−1^, the capacitive contribution of PS‐0.5NHC reaches 88.72% (Figure 4c). Compared to other anodes (Figure S25), PS‐0.5NHC exhibits a consistently higher capacitive contribution across different scan rates (Figure 4e). The essence of this phenomenon lies in the fact that nitrogen doping introduces a large number of N‐5 defects and surface active sites into 0.5NHC, providing ample reversible sodium‐replenishment sites for presodiation. Following presodiation, the supplemented Na^+^ preferentially reacts with the electrolyte to preconstructed a dense SEI film. This SEI passivates irreversible defects on the electrode surface (reducing consumption from side reactions) while preventing continuous contact between the electrolyte and active material. Consequently, it significantly accelerates the interfacial transport kinetics of Na^+^ and enhances the proportion of capacitive contribution.

The Na^+^ diffusion coefficient (D_Na+_) of the electrode during charging and discharging is calculated using the GITT test, as shown in Equation (3) [56].

(3) $$D_{Na^{+}}=\frac{4}{\pi \tau }{\left(\frac{m_{B}V_{M}}{M_{B}S}\right)}^{2}{\left(\frac{\Delta E_{s}}{\Delta E_{t}}\right)}^{2}$$

In the equation, m_B_, V_M_, M_B_, S, ΔE_s_, ΔE_t_, and τ represent the mass of active material, molar volume, molecular weight, electrode geometric area, steady‐state potential change, transient potential change, and pulse duration, respectively. As shown in Figure 4f, the diffusion coefficients D exhibit consistent trends across the four electrode types. However, the logarithmic diffusion coefficient (logD) of the PS‐0.5NHC anode remains within the highest range (−9.67 to −8.70 cm^2^ s^−1^) throughout the entire discharge process. This contrasts with HC (logD = −10.3 to −9.3 cm^2^ s^−1^), 0.5NHC (logD = −9.99 to −9.11 cm^2^ s^−1^), and PS‐HC (logD = −10.05 to −9.22 cm^2^ s^−1^). This result further confirms that the defect structures generated by nitrogen doping provide efficient sodium‐supplementation pathways for presodiation. The supplemented Na^+^ not only serves as a reversible sodium source but also participates in constructing the SEI film with high ionic conductivity. This SEI film synergizes with the expanded carbon layer spacing (d_002_ = 0.384 nm) to provide a low‐resistance pathway for bulk Na^+^ diffusion, ultimately endowing PS‐0.5NHC with superior sodium storage kinetics.

To investigate the synergistic effect of nitrogen doping and presodiation on electrode interface impedance, we employed in situ EIS to dynamically monitor the entire charge–discharge process of PS‐0.5NHC and HC electrodes, yielding corresponding Nyquist plots (Figure S26a,b). Comparative analysis reveals that the PS‐0.5NHC electrode consistently exhibits lower charge transfer resistance (*R*_ct_) and a more stable trend of interfacial impedance growth throughout the entire charge–discharge cycle. This directly corroborates the stability of its electrode/electrolyte interface structure and efficient charge transfer kinetics—a finding consistent with the high capacitance contribution rate observed in CV testing and the high Na^+^ diffusion coefficient in GITT testing. Due to the overlapping impedance responses of different kinetic processes in the Nyquist plot of EIS (e.g., the impedance of the SEI film and charge transfer impedance are often conflated in the mid‐frequency region), we performed a distribution of relaxation times (DRT) inversion calculation on the in situ EIS data to more intuitively distinguish the contributions of each process. The resulting relaxation time distribution curves (γ(τ) vs. τ) are presented in Figure 4g and Figure S27. In DRT analysis, each γ peak corresponds to an independent electrochemical relaxation process: the peak position (relaxation time τ) reflects the kinetic rate (the smaller τ, the faster the process response); the peak area quantifies the impedance contribution of this process (the larger the area, the higher the proportion of impedance). Specifically, γ peaks with relaxation times in the 10^−5^ to 10^−4^ s range correspond to the high‐frequency response in the Nyquist plot and can be clearly attributed to the ion transport impedance of the SEI film (R_SEI_) [57]. Throughout the discharge process (Na^+^ intercalation stage, Figure 4h,i), the position of the γ peak for PS‐0.5NHC shifted only slightly to the right, indicating minimal changes in the ion transport kinetics of the SEI layer. Furthermore, its R_SEI_ consistently remained at a low level (< 10 Ω), demonstrating that the preconstructed SEI layer maintained structural stability during Na^+^ intercalation without significant disruption or restructuring. In contrast, HC exhibits a larger initial τ value for this γ peak (∼1.7 × 10^−4^ s). Although R_SEI_ decreases slightly during discharge, it remains significantly higher than that of PS‐0.5NHC — this is associated with the poor stability of the SEI film on HC surface due to the lack of presodiation regulation. Repeated Na^+^ intercalation leads to continuous rupture and repair of the SEI, thereby increasing ion transport resistance. Repeated Na^+^ intercalation leads to continuous rupture and repair of the SEI, thereby increasing ionic transport resistance. The results from in situ EIS combined with DRT inversion further confirm that the defect sites introduced by nitrogen doping provide efficient sodium‐supplementation pathways for presodiation, enabling PS‐0.5NHC to form a highly stable SEI layer with superior ionic conductivity during the initial cycling stage. This SEI layer synergizes with the optimized carbon layer structure to ensure stable interfacial impedance and efficient charge transfer during Na^+^ insertion/extraction. This core interfacial mechanism underlies the kinetic advantage of PS‐0.5NHC over HC and single‐modified samples (0.5NHC, PS‐HC). To thoroughly elucidate the Na^+^ storage mechanism of the PS‐0.5NHC anode, we employed in situ Raman spectroscopy to track its structural evolution during charge–discharge cycles, with results shown in Figure 4j–l. At the onset of discharge, the intensity of the D peak in the Raman spectrum gradually diminishes, while the position and intensity of the G peak remain largely unchanged. This occurs because nitrogen‐doped defects (N‐5 sites) and edge active sites on the PS‐0.5NHC surface are preferentially adsorbed by Na^+^, thereby suppressing the vibrational degrees of freedom of carbon rings and leading to a weakening of defect‐related scattering signals. As the discharge proceeded, the G peak exhibited a redshift (from 1580 to 1557 cm^−1^). This shift is directly related to the structural changes induced by Na^+^ intercalation into the graphite domains. The intercalation of Na^+^ ions extends the bond length between carbon atoms and reduces the bond force constant (k). According to the principles of Raman scattering, this reduction in the force constant leads to a decrease in vibrational frequency, thereby manifesting as the observed red shift of the G peak to a lower wavenumber. When the discharge reaches 0.1 V, the D and G peaks essentially vanish. This occurs because Na^+^ ions have filled the nanopores, forming quasi‐metallic sodium domains that occupy the π^*^ antibonding orbitals. This suppresses the conjugated vibration of the C─C bonds, resulting in a decrease in peak intensity until complete disappearance in the Raman spectrum [8, 58]. During charging, peaks D and G gradually return to their original states, demonstrating the reversible insertion/desorption of Na^+^ in PS‐0.5NHC.

In summary, the PS‐0.5NHC anode exhibits outstanding comprehensive electrochemical performance: it not only possesses high reversible sodium storage capacity and excellent rate capability but also achieves long‐term cycling stability and rapid ion diffusion kinetics. This performance advantage is not merely the simple sum of nitrogen doping and presodiation, but rather results from the synergistic effects of structural optimization and interfacial regulation.

To clarify how N‐doped HC with abundant active sites affects the structural characteristics of preconstructed SEI during presodiation and its stability after cycling, we systematically compared the electrode‐electrolyte interface properties of PS‐0.5NHC (N‐doped presodiated HC) and PS‐HC (undoped presodiated HC). The interface regulation mechanism was revealed by a combination of HRTEM, EDS, and XPS.

**FIGURE 5:**
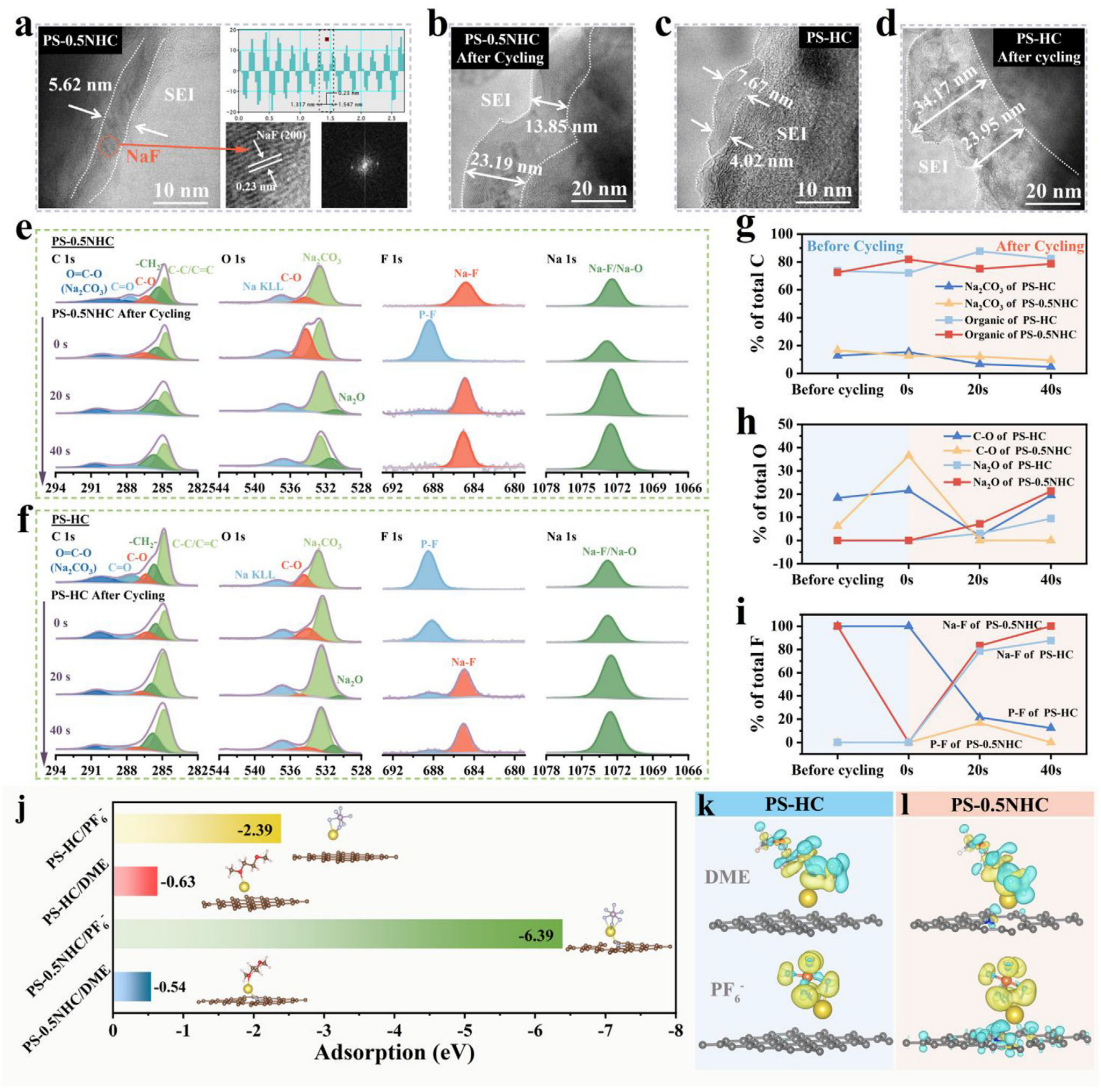
HRTEM images of (a,b) PS‐0.5NHC and (c,d) PS‐HC anodes before and after cycling. C 1s, O 1s, F 1s, and Na 1s XPS spectra of (e) PS‐0.5NHC and (f) PS‐HC anodes before and after cycling. The proportion of SEI components calculated from the (g) C 1s, (h) O 1s, and (i) F 1s spectra. (j) Calculated adsorption energies of PS‐0.5NHC and PS‐HC for the DME solvent molecule and the PF_6_^−^ anion. Differential Charge density isosurfaces for (k) PS‐0.5NHC and (l) PS‐HC with DME and PF_6_^−^.

Direct observation of the SEI microstructure on both electrodes before and after cycling via HRTEM, combined with EDS plane scanning analysis of elemental spatial distribution. Before cycling (preconstructed SEI stage), the PS‐0.5NHC electrode surface exhibited a broadly uniform SEI film (Figure 5a) with a thickness of approximately 5–6 nm. Rapid Fourier Transform (RFT) analysis revealed the 200 crystal plane of NaF (interlayer spacing 0.23 nm), confirming its enrichment with highly ion‐conductive inorganic phases [59]. EDS surface scanning results (Figures S28 and S29) reveal a uniform distribution of fluorine (F) elements with a content of 4.22%. In contrast, the preconstructed SEI layer of PS‐HC exhibits no distinct NaF signal (Figure 5c), containing only 0.62% F elements. Additionally, localized exposed areas are present within the membrane layer, indicating poor uniformity. After ten cycles (SEI stability verification): The SEI of PS‐0.5NHC maintained a smooth surface with uniform thickness (∼20 nm) (Figure 5b), showing no significant cracking or localized thickening, while its F content remained substantially higher than that of PS‐HC (Figures S30 and S31). In contrast, the SEI of PS‐HC exhibited a rough surface with a loose internal structure, and its thickness abruptly increased to 34 nm (Figure 5d). This indicates that nitrogen doping introduces highly reversible active sites into HC, enhancing cation supply and reaction uniformity during the presodiation stage. This promotes NaF formation, making the resulting SEI more resistant to restructuring during cycling and exhibiting significantly superior structural stability compared to the undoped sample.

To further analyze the chemical composition and interlayer distribution of the SEI, high‐resolution XPS measurements of C 1s, O 1s, F 1s, and Na 1s were performed on both electrodes before and after cycling, combined with Ar^+^ sputter depth profiling. For the PS‐0.5NHC electrode before cycling, the C 1s spectra at 284.85 eV (C─C/C═C), 285.77 eV (‐CH_2_‐), 286.87 eV (C─O), and 288.4 eV (C═O) correspond to organic components such as R‐O‐Na generated from the decomposition of the DME electrolyte. Simultaneously, peak signals at 290.7 eV (O═C─O), 532.53 eV (Na_2_CO_3_), 685.04 eV (Na‐F), and 1072.58 eV (Na‐O/Na‐F) confirm the presence of inorganic phases Na_2_CO_3_, Na_2_O, and NaF. In contrast, the preconstructed SEI on the PS‐HC electrode only detected the inorganic phases Na_2_CO_3_ and Na_2_O, along with the organic phase R‐O‐Na, without any NaF inorganic component. This result aligns with HRTEM observations. The F 1s peak at 688.23 eV (P─F) corresponds to organic fluorides generated from PF_6_^−^ decomposition. Through Ar^+^ sputtering depth profiling, the composition of the SEI at different depths on the cycled PS‐0.5NHC and PS‐HC electrodes was measured (Figure 5e–i). As sputtering time (depth) increased, the inorganic content within the SEI of PS‐0.5NHC gradually rose. Meanwhile, P─F and C─O bond energies remained detectable in the SEI inner layer of PS‐HC, with organic components predominantly distributed in the middle layer. The distribution trends of Na_2_CO_3_ and Na_2_O in the SEI components of PS‐0.5NHC and PS‐HC anodes are identical. Na_2_CO_3_ exhibits a relatively uniform distribution within the SEI layer, while Na_2_O is primarily distributed in the inner layer of the SEI. However, with increasing sputtering depth, the deconvolution results of the F 1s peak show significant differences, which are related to the preconstructed SEI layer.

To elucidate the regulatory mechanism of N doping on the PF_6_^−^ adsorption capability of presodiated HC, DFT theoretical calculations were performed to systematically analyze the adsorption energies between PS‐0.5NHC, PS‐HC, and key electrolyte components (the solvent DME and the anion PF_6_^−^). Furthermore, the differential charge densities for the four systems (PS‐0.5NHC/DME, PS‐0.5NHC/PF_6_^−^, PS‐HC/DME, PS‐HC/PF_6_^−^) were calculated, with the results presented in Figure 5j–l. The adsorption energy data reveal that both materials exhibit relatively weak and similar adsorption energies toward the solvent molecule DME, indicating that nitrogen doping does not significantly alter the adsorption characteristics for solvent molecules. However, for the PF_6_^−^ anion, the adsorption energy (ΔE_ad_) of PS‐0.5NHC (−6.39 eV) is significantly more negative than that of PS‐HC (−2.39 eV), demonstrating that the PS‐0.5NHC significantly enhances the adsorption of PF_6_^−^ by strengthening the adsorption of Na^+^ via the introduced N‐5 defects (Figure 5j). Adsorption models from other perspectives are presented in Figure S32. The differential charge densities further corroborate this conclusion. In the PS‐0.5NHC/PF_6_^−^ system, a pronounced electron density accumulation is observed around PF_6_^−^, accompanied by significant electron transfer between the anion and the active sites (N‐5 sites) on the PS‐0.5NHC surface, indicating a strong electronic interaction (Figure 5k). In contrast, within the PS‐HC/PF_6_^−^ system, the electron density distribution around PF_6_^−^ is relatively sparse, and the electron transfer with the hard carbon surface is weaker than in the former case, which can be attributed to the weaker adsorption capability of HC toward Na^+^ (Figure 5l). Conversely, for the interaction systems involving DME and the two hard carbons (PS‐0.5NHC/DME, PS‐HC/DME), no significant electron density enrichment or strong electron transfer characteristics are observed in either case (Figure S33). Therefore, presodiation enriched with active sites enhances the adsorption of PF_6_^−^ by increasing the supply of cations (Na^+^), thereby providing sufficient reaction conditions for the formation of NaF. The inner layer of the SEI, containing inorganic compounds like NaF, ensures high ionic conductivity while its superior mechanical strength and modulus effectively support the SEI structure. This significantly reduces the continuous thickening of the SEI caused by the ongoing reduction and decomposition of electrolyte on the electrode surface. The organic components in the outer layer of the SEI buffer stresses generated by volume expansion/contraction of electrode materials during charge–discharge cycles through their superior flexibility, preventing overall brittle cracking of the SEI [60]. This SEI structure is one of the key factors enabling high‐performance HC anodes to achieve long cycle life.

**FIGURE 6:**
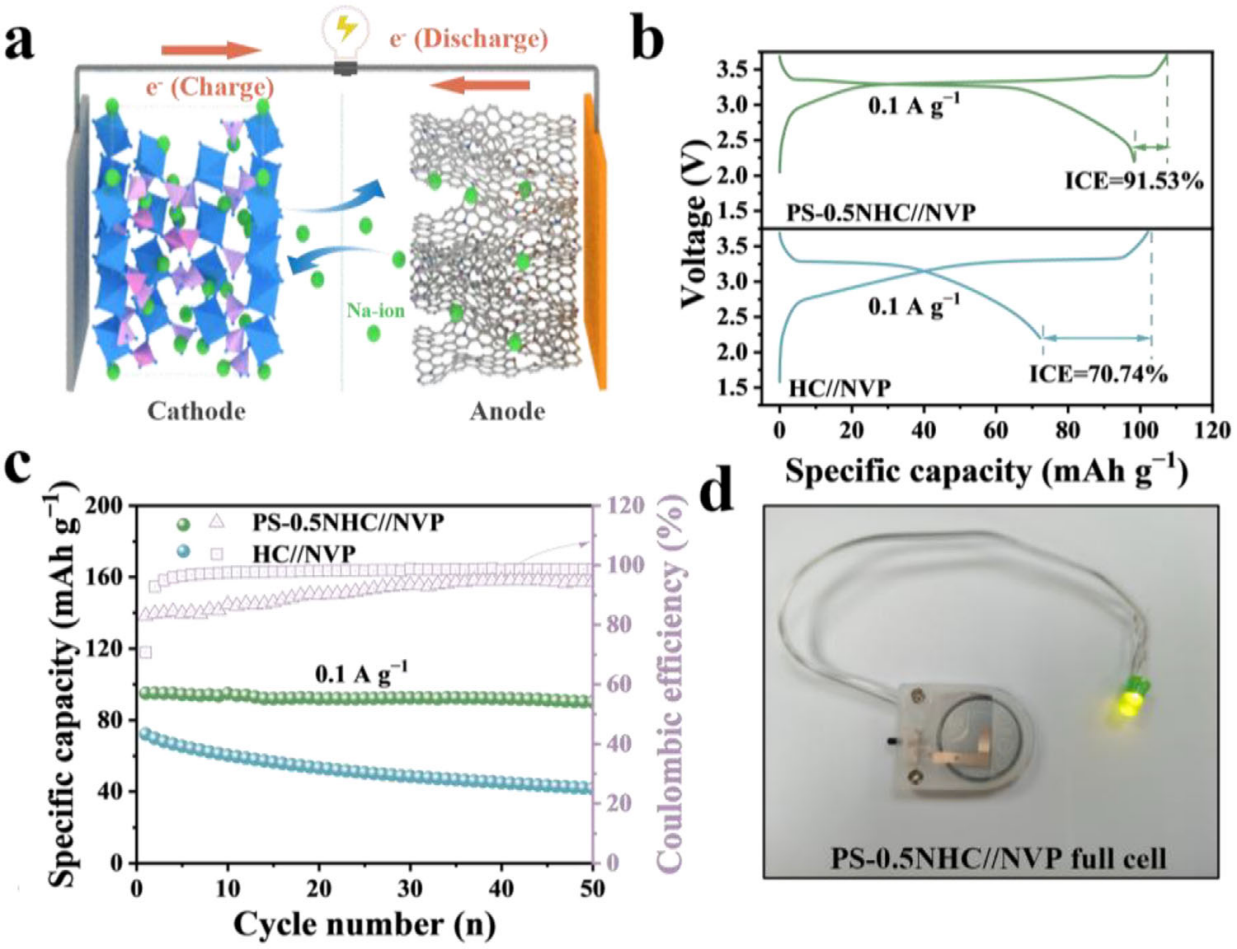
(a) Schematic diagram of PS‐0.5NHC//NVP. (b) Initial GCD profiles and (c) cycling performance of PS‐0.5NHC//NVP and HC//NVP full cells at 0.1 A g^−1^. (d) LED bulb lit by the PS‐0.5NHC//NVP full‐cell.

To evaluate the practical application potential of the PS‐0.5NHC anode, a full‐cell was constructed by combining the PS‐0.5NHC anode with a NVP (Figure 6a). Figure 6b compares the initial GCD performance of PS‐0.5NHC//NVP and HC//NVP full cells within the voltage range of 2.2–3.7 V. At a current density of 0.1 A g^−1^, the PS‐0.5NHC//NVP full‐cell demonstrated an ICE as high as 91.53%, while achieving a reversible specific capacity of 98.28 mAh g^−1^. This indicates that Na^+^ supplied by the cathode can be efficiently utilized for reversible sodium storage at the anode. The HC//NVP full‐cell exhibited an ICE of only 70.74%, with a reversible specific capacity of just 72.23 mAh g^−1^. This is attributed to the significant consumption of Na^+^ during the formation of the SEI in the first cycle of cycling, as well as the irreversible trapping of Na^+^ by defects. Furthermore, after 50 cycles at a current density of 0.1 A g^−1^ (Figure 6c), the PS‐0.5NHC//NVP exhibited an impressive capacity retention of 95.06%, demonstrating outstanding cycling performance. Moreover, compared with other reported full‐cell systems, the PS‐0.5NHC//NVP shows better comprehensive performance (Figure S34), highlighting the effectiveness of our synergistic strategy. Simultaneously assembled PS‐0.5NHC//NVP full‐cell could illuminate an LED bulb (Figure 6d), indicating the practical application potential of this method‐modified HC.

## 3 Conclusion

This study addresses the issues of significant first‐cycle sodium loss and poor interfacial stability in HC anodes for SIBs by proposing a “N‐doping/presodiation” synergistic regulation strategy. It systematically investigates the sodium‐replenishment mechanism and electrochemical performance of PS‐0.5NHC. Results demonstrate that the reversible active sites introduced by N‐doping exhibit dual effects: On one hand, it enhances the reversible capacity (301.34 mAh g^−1^ @ 0.03 A g^−1^); on the other hand, it improves the presodiation efficiency (sodium supplementation content of 7.4%, representing a 48.6% increase compared to HC), which strengthens the adsorption of PF_6_^−^ by PS‐0.5NHC. This promotes the deep reduction of PF_6_^−^, and the reduced products combine with Na^+^ to form NaF, thereby constructing a stable SEI with an inorganic‐rich inner layer. Benefiting from the exceptional structural stability of SEI, PS‐0.5NHC maintained a high capacity retention rate of 90.6% after 1000 cycles at 1.5 A g^−1^, demonstrating outstanding long‐cycle performance. In the full‐cell system, the device assembled with PS‐0.5NHC as the anode and Na_3_V_2_(PO_4_)_3_ as the cathode achieved an ICE of 91.53% at 0.1 A g^−1^, with a reversible capacity of 98.28 mAh g^−1^. After 50 cycles, the capacity retention rate remained at 95.06%, further validating the practical value of this strategy. This study provides innovative insights for optimizing HC anode interfaces and enhancing the overall performance of SIBs.

## Author Contributions

**Hua Lin**: Methodology, Formal analysis, Investigation. Writing – original draft. **Wenxing Miao**: Methodology, Software, Formal analysis. **Yanrong Shi**: Investigation, Methodology. **Guangyi Mao**: Investigation, Methodology. **Ding Ding**: Investigation, Methodology. **Jian Weng**: Resources, Funding acquisition, Supervision. **Zhongxiong Fan**: Resources, Funding acquisition, Supervision. **Qingchi Xu**: Resources, Funding acquisition, Supervision. **Jun Xu**: Resources, Funding acquisition, Writing – review and editing.

## Conflicts of Interest

The authors declare no conflicts of interest

## Supporting information




**Supporting File**: advs75460‐sup‐0001‐SuppMat.docx.

## Data Availability

The data that support the findings of this study are available from the corresponding author upon reasonable request.
