# Supplementary material for: N‐Doping Activated Presodiation Enhances Sodium‐Ion Provision in Hard Carbon Anodes
Source: Adv Sci (Weinh). 2026 Jun 22:e75460. Online ahead of print. doi: 10.1002/advs.75460 (PMC13335756; doi:10.1002/advs.75460)
Supplement: Supplementary file 1 — Supporting File: advs75460‐sup‐0001‐SuppMat.docx. [file ADVS-9999-e75460-s001.docx]

Supporting Information

**N-Doping Activated Presodiation Enhance Sodium-ion Provision in Hard Carbon Anodes**

*Hua Lin^1^, Wenxing Miao^1^, Yanrong Shi^2,3^, Guangyi Mao^1^, Ding Ding^1^, Jian Weng^4^*, Zhongxiong Fan^2,3^*, Qingchi Xu^1^*, Jun Xu^1^**

E-mail: jweng@xmu.edu.cn (J. Weng); fanzhongxiong@xju.edu.cn (Z. Fan); xuqingchi@xmu.edu.cn (Q. Xu); xujun@xmu.edu.cn (J. Xu).

^1^ Department of Physics, Research Institute for Biomimetics and Soft Matter, Fujian Provincial Key Laboratory for Soft Functional Materials, Xiamen University, Xiamen 361005, China.

^2^ School of Pharmaceutical Sciences, Institute of Materia Medica, Xinjiang University, Urumqi 830017, China.

^3^ Xinjiang Uygur Autonomous Region, State Key Laboratory of Chemistry and Utilization of Carbon Based Energy Resources, Xinjiang University, Urumqi 830017, China.

^4^ The Higher Educational Key Laboratory for Biomedical Engineering of Fujian Province, Research Center of Biomedical Engineering of Xiamen, Department of Biomaterials College of Materials, Xiamen University, Xiamen 361005, China.

### Materials

Sodium metal (Na, 99.5%) and hydrochloric acid (HCl, 37–38 wt%) were purchased from Sinopharm Chemical Reagent Co., Ltd. (SCRC, Shanghai, China). Urea and biphenyl (Bp, 98%) were obtained from Macklin Biochemical Co., Ltd. (Shanghai, China). Acetylene black, sodium carboxymethyl cellulose (CMC), polyvinylidene fluoride (PVDF), Super-P, vanadium sodium phosphate (Na_3_V_2_(PO_4_)_3_) and N-methylpyrrolidone (NMP) were supplied by Kejing Materials Technology Co., Ltd. (Hefei, China). 1,2-Dimethoxyethane (DME) and sodium hexafluorophosphate (NaPF_6_) electrolyte (1.0 mol L^−1^ NaPF_6_ in pure DME, 100 vol%) were purchased from Duoduo Reagent Co., Ltd. (Chengdu, China). All chemicals were used as received without further purification unless otherwise stated.

### Experimental section

#### Preparation of HC precursor (HCP)

The natural coconut shells were cut into small pieces of 1–3 cm, cleaned, and then dried in an oven at 120 °C for 12 hours to remove surface impurities and moisture. In a tube furnace under nitrogen atmosphere, the temperature was raised to 400 °C at a rate of 5 °C/min and held for 2 hours for low-temperature carbonization, so as to remove volatile organic compounds from the coconut shells. The coconut shells after low-temperature carbonization were crushed into powder using a high-speed pulverizer, and the powder was collected after sieving through an 800-mesh sieve for purification. 10 g of the powder was added to a 100 mL beaker containing 38 mL of deionized water. Subsequently, 2 mL of HCl (37 wt%) was slowly added dropwise, and continuous magnetic stirring was conducted at room temperature (300 rpm, 4 hours). After stirring, the solid product was separated by centrifugation (11,000 rpm, 10 minutes), and then thoroughly centrifuged and washed repeatedly with deionized water three times until the pH value of the supernatant became neutral (pH ≈ 7). The washed sample (HC precursor) was transferred to a vacuum oven and dried at 80 °C for 24 hours to obtain the HC precursor (HCP).

#### Preparation of XNHC, HC

#### As shown in Fig. S1a, pure hard carbon anode materials were first prepared by pyrolyzing the carbon precursor (HCP) at different temperatures (1000 °C, 1200 °C, 1400 °C) under a high-purity nitrogen atmosphere (heating rate: 2 °C/min, holding time: 2 h), denoted as HC-1000, HC-1200, and HC-1400, respectively. For the sake of simplicity, HC-1200 (pyrolyzed from HCP at 1200 °C without urea addition) is subsequently referred to as "HC" in this study.

#### For the synthesis of nitrogen-doped hard carbon materials, urea (as the nitrogen source) and the pristine carbon precursor (HCP) were fully ground and uniformly mixed at different mass ratios (0.2, 0.5, 0.8). The mixed precursors were then pyrolyzed under the identical atmosphere conditions (high-purity N_2_, heating rate: 2 °C/min, holding time: 2 h) but fixed at a final temperature of 1200 °C. Finally, nitrogen-doped hard carbon anode materials with a pseudo-graphitic structure were obtained, designated as "XNHC" (where 'X' represents the mass ratio of urea to HCP: 0.2NHC, 0.5NHC, 0.8NHC).

#### Preparation of PS-0.5NHC and PS-HC anodes

The presodiated solution was prepared according to the method reported by Liu et al.^[1]^ 11.5 mg of Na and 77.1 mg of Bp were dissolved in 1 mL of DME, and stirred for 30 minutes to obtain a uniform dark blue presodiated solution, named Na-BP-DME. The as-prepared 0.5NHC and HC anode sheets (see Section 1.6. for detailed preparation method of the anode sheets) were immersed in the Na-BP-DME solution for a certain period of time (**Fig. S1b**). After being taken out, the anode sheets were rinsed with DME solvent, then vacuum-dried for subsequent use. (The above operations must be carried out in a glove box, with the oxygen and water contents controlled below 0.5 ppm.)

### Physical Characterization

The morphology, microstructure, and elemental distribution of the samples were characterized using scanning electron microscopy (SEM, SIGMA-HD), high-resolution transmission electron microscopy (HRTEM, FEI Tecnai G2 F30, USA), and energy-dispersive spectroscopy (EDS, attached to SIGMA-HD), respectively. X-ray diffraction (XRD) measurements were performed on a Bruker-axs XRD diffractometer with Cu Kα radiation (λ = 1.5418 Å) in the 2θ range of 10°–70° to analyze the crystal structure of the samples. The surface chemical composition of the samples was investigated via X-ray photoelectron spectroscopy (XPS, ESCALAB Xi+, Thermo Fisher Scientific). N2 adsorption-desorption isotherms were measured using a Micromeritics TriStar II Plus analyzer. The specific surface area (SSA) of hard carbon samples was calculated by the Barrett-Emmett-Teller (BET) method, while the pore size distribution was analyzed using the Barrett-Joyner-Halenda (BJH) model based on the desorption branch of the isotherms. Raman spectra and in-situ Raman tests were acquired using a Labram HR Evolution Raman spectrometer with a 532 nm excitation laser (laser power: 100 mW).

### Electrochemical Characterization

The anode for SIBs was fabricated by homogeneously mixing the active material (HC, XNHC), conductive agent Super-P, and binder CMC in a mass ratio of 8:1:1 in deionized water via magnetic stirring. The resulting slurry was coated onto a copper foil current collector using a doctor-blade method and vacuum-dried at 60°C for 12 h. For the cathode, Na_3_V_2_(PO_4_)_3_, Super P, and PVDF binder were blended in the same mass ratio (8:1:1) in NMP solvent to form a uniform slurry, which was then coated onto an aluminum foil current collector and dried at 80°C for 12 h under vacuum. CR2032 coin cells were assembled in an argon-filled glove box using 1.0 mol L^−1^ NaPF_6_ in DME as the electrolyte and a glass fiber separator (Whatman GF/D). Metallic sodium foil served as the counter electrode in half-cell configurations. For full-cell assemblies, the cathode and anode were capacity-balanced with an N/P ratio of 1.05–1.13. Galvanostatic charge-discharge (GCD) tests were performed within voltage ranges of 0.01–3.0 V and 2.2–3.7 V using a Neware battery testing system. Cyclic voltammetry (CV) and electrochemical impedance spectroscopy (EIS) measurements were conducted on a CHI660E electrochemical workstation, with EIS parameters set to a frequency range of 0.01–10^5^ Hz and an amplitude of 5 mV. Galvanostatic intermittent titration technique (GITT) tests employed a 0.03 A g^−1^ pulse current for 600 s, followed by a 1.5 h relaxation interval.

### Density functional theory (DFT) calculation method

Computational methods. The Vienna Ab Initio Package (VASP) was employed to perform all the density functional theory (DFT) calculations.^[2-3]^ The projected augmented wave (PAW) was applied to describe the ion-electron interaction and electron exchange, and corresponding energies were obtained within the generalized gradient approximation in the Perdew-Burke-Ernzerh (GGA-PBE) formalism.^[4]^ A plane wave basis with a kinetic energy cutoff of 500 eV was utilized. Partial occupancies of the Kohn-Sham orbitals were allowed using the Gaussian smearing method with a width of 0.03 eV. The electronic energy was considered self-consistent when the energy change was smaller than 10-6 eV, and a geometry optimization was considered convergent when the force change was smaller than 0.01 eV/Å. The Grimme’s DFT-D3 approach was used to treat the dispersion interactions. The charge density difference was calculated as ∆ρ = ρtotal - ρA - ρB, where the ρtotal was the charge density of binding systems, ρA or ρB was the sub charge density. Further, the adsorption energy (Eads) was defined as Eads = Ead/sub -Ead -Esub, where Ead/sub, Ead, and Esub were total energies of the optimized adsorbate/substrate system, adsorbate in the structure, and clean substrate, respectively.


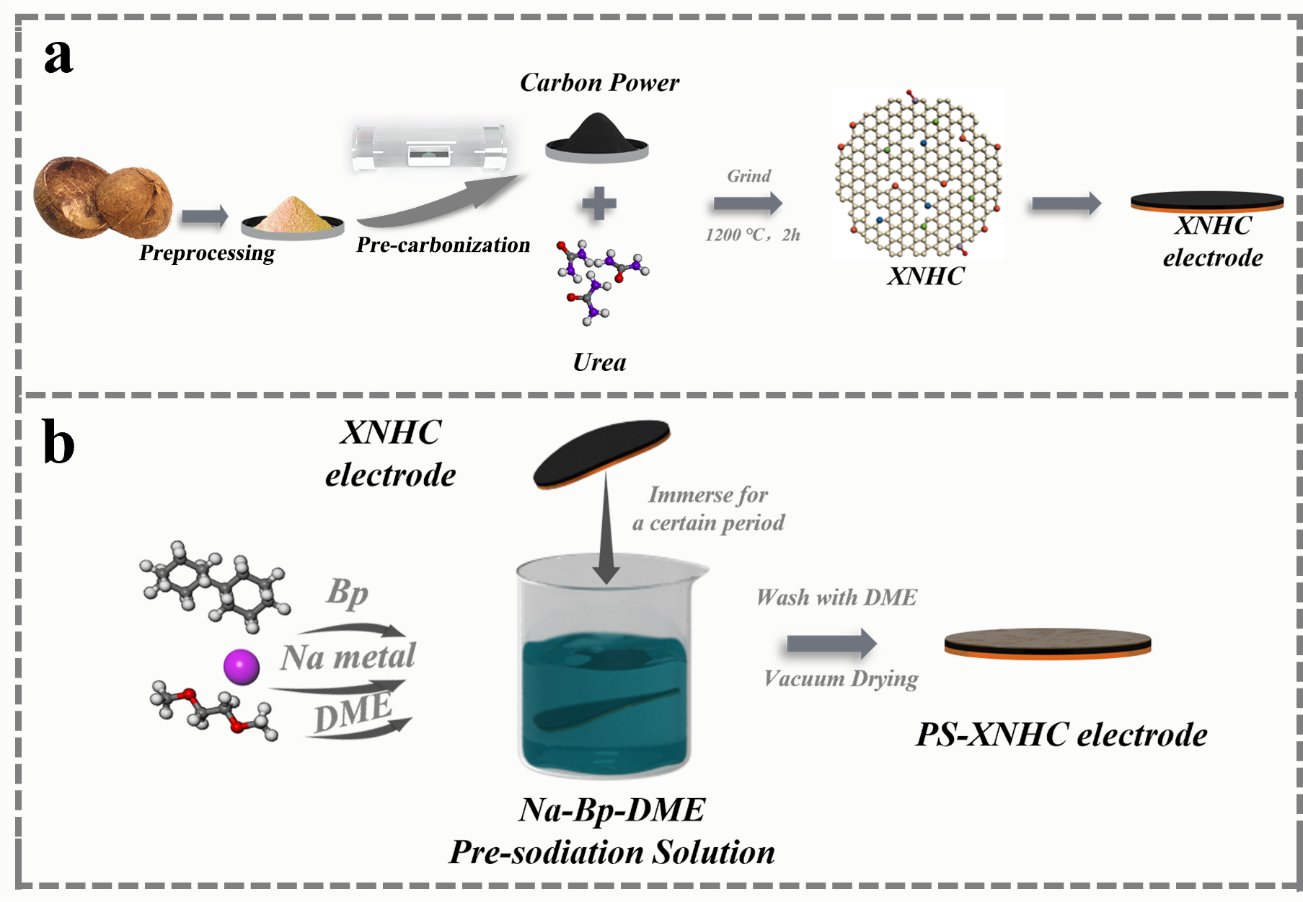


Fig. S1 a Synthesis process of nitrogen-doped hard carbon (PS-XNHC) anode with rich active sites. b Presodiation treatment for PS-XNHC anode.


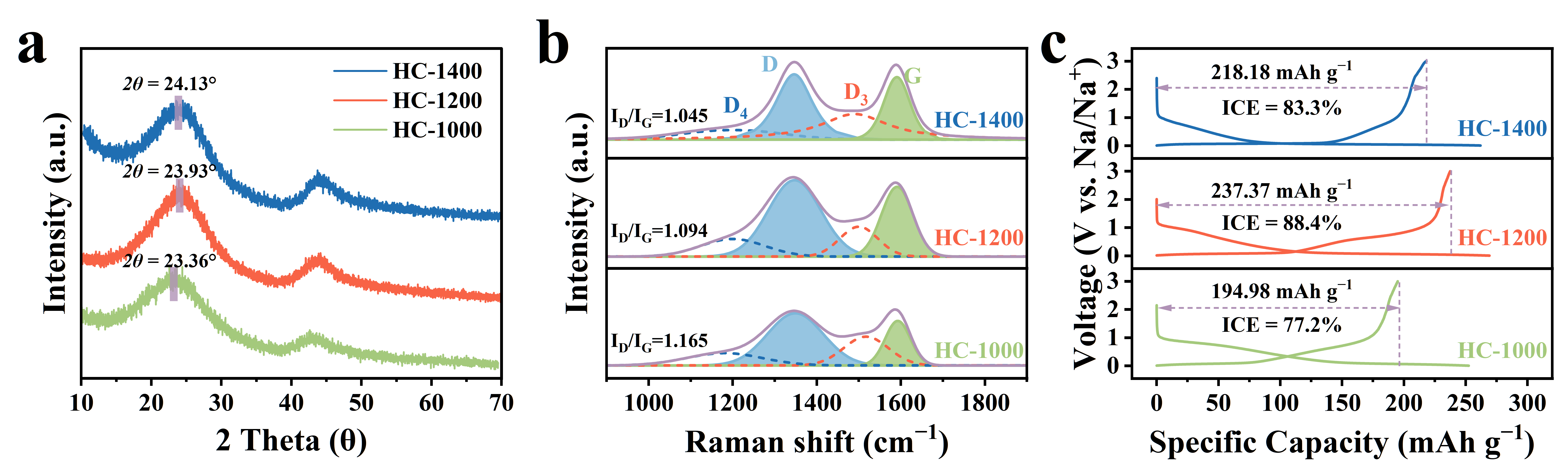


**Fig. S2 a** XRD spectra **b** Raman spectra, and **c** Initial GCD curves (at 0.03 A g^−1^) of HC-1000, HC-1200 (HC), and HC-1400.

To provide a clear rationale for selecting 1200 °C as the pyrolysis temperature, we have conducted a systematic investigation by preparing additional hard carbon samples at 1000 °C and 1400 °C (denoted as HC-1000 and HC-1400) and compared their structural and electrochemical properties with the HC-1200 sample.

XRD analysis (**Fig. S2a**) shows that the (002) diffraction peak shifts from 23.36° for HC-1000 to 24.13° for HC-1400, corresponding to a gradual decrease in interlayer spacing from 0.381 nm to 0.369 nm. The interlayer spacing of HC-1200 is 0.372 nm, representing a moderate value. Raman spectroscopy (**Fig. S2b**) reveals that the I_D_/I_G_ ratio decreases from 1.165 (HC-1000) to 1.045 (HC-1400), indicating reduced defect density with increasing temperature. HC-1200 exhibits an I_D_/I_G_ ratio of 1.094, which offers a balance between sufficient active sites and structural order.

Electrochemical evaluation (**Fig. S2c**) demonstrates that HC-1000 delivers a reversible capacity of only 194.98 mAh g^−1^ with a low ICE of 77.2%, attributed to excessive defects that promote irreversible Na⁺ trapping and side reactions. HC-1400, despite its higher graphitization degree, suffers from excessively contracted interlayer spacing (0.369 nm), which impedes Na^+^ intercalation kinetics and results in a limited reversible capacity of 203.18 mAh g^−1^. In contrast, HC-1200 achieves a high reversible capacity of 237.37 mAh g^−1^ and an ICE of 88.4%. Moreover, at a temperature of 1400 °C, the nitrogen doping efficiency decreases sharply, and energy consumption along with production costs increase significantly, hindering practical scalability for large-scale applications. By comparison, a temperature of 1200 °C is sufficient to create a stable carbon framework while retaining a substantial number of defects. This is crucial for our strategy, as we specifically aim to introduce and preserve a high concentration of pyrrolic-N (N-5) defects.

Therefore, 1200 °C was selected as the optimal carbonization temperature for subsequent nitrogen doping to prepare the 0.5NHC material, which further benefits from expanded interlayer spacing (0.384 nm) and abundant N-5 defects introduced by urea doping.


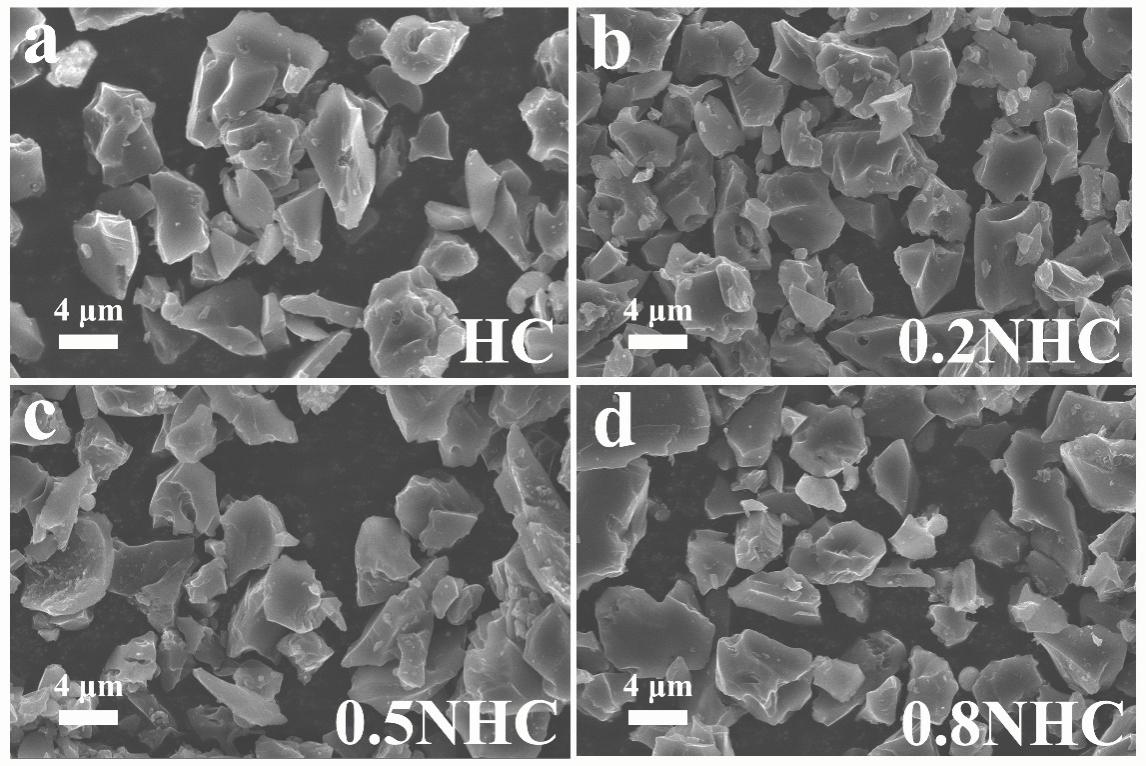


Fig. S3 SEM images of a HC, b 0.2NHC, c 0.5NHC and d 0.8NHC.


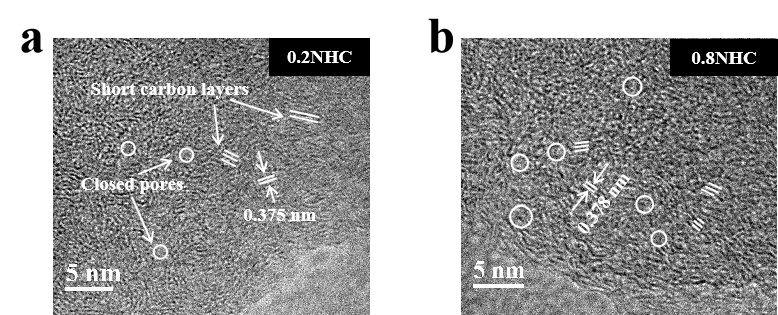


Fig. S4 HRTEM images of a 0.2NHC and b 0.8NHC.


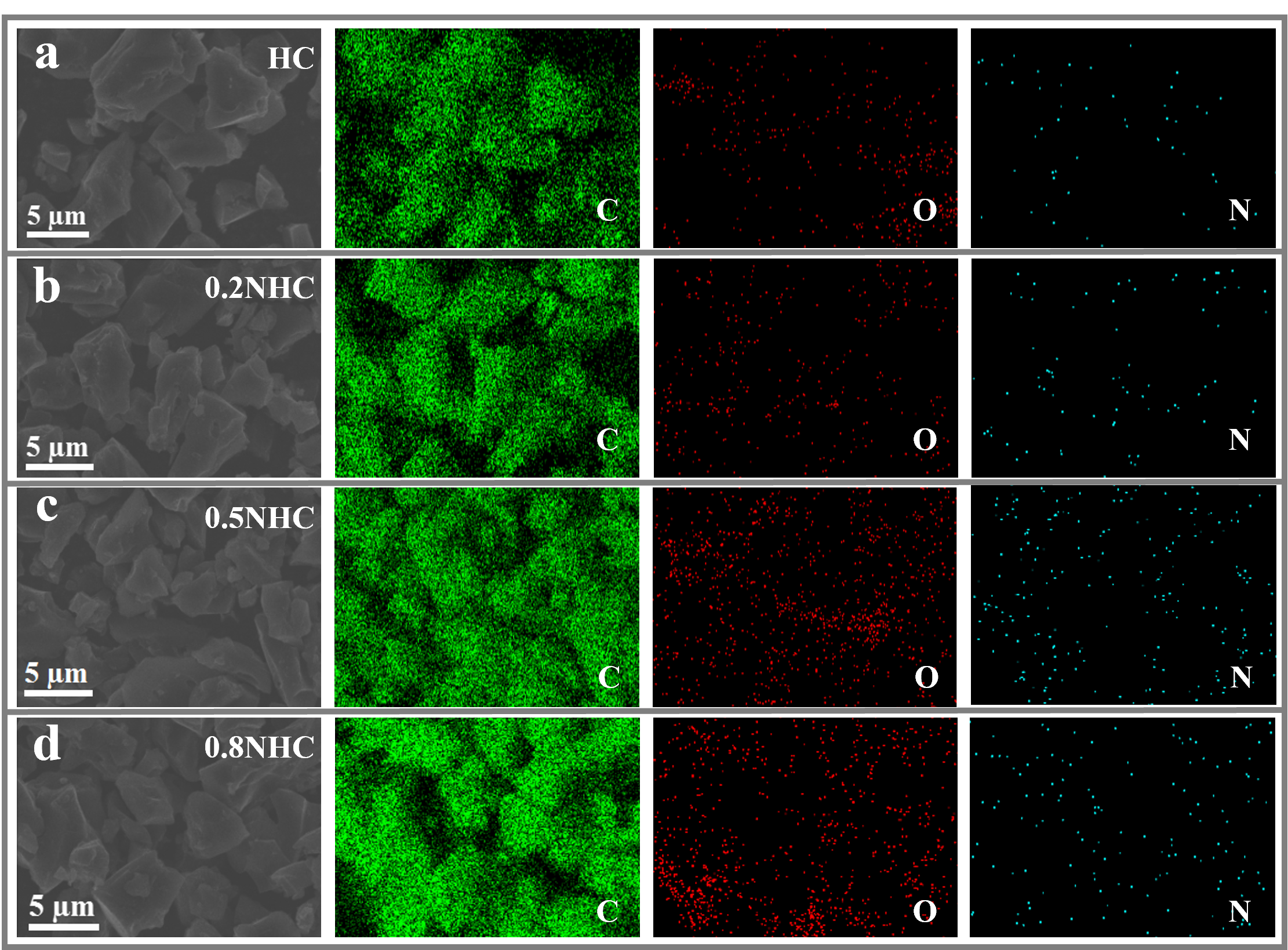


Fig. S5 SEM-EDS mapping of HC, 0.2NHC, 0.5NHC, and 0.8NHC. Overall morphology and distribution of C, O, Na elements in a HC, b 0.2NHC, c 0.5NHC, and d 0.8NHC.


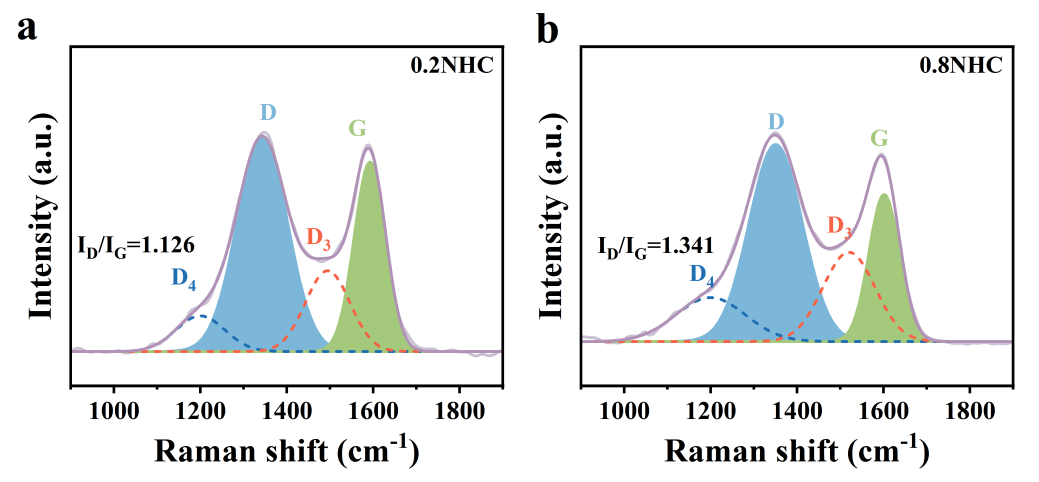


Fig. S6 Raman spectrums of a 0.2NHC and b 0.8NHC obtained with a 532 nm laser excitation.


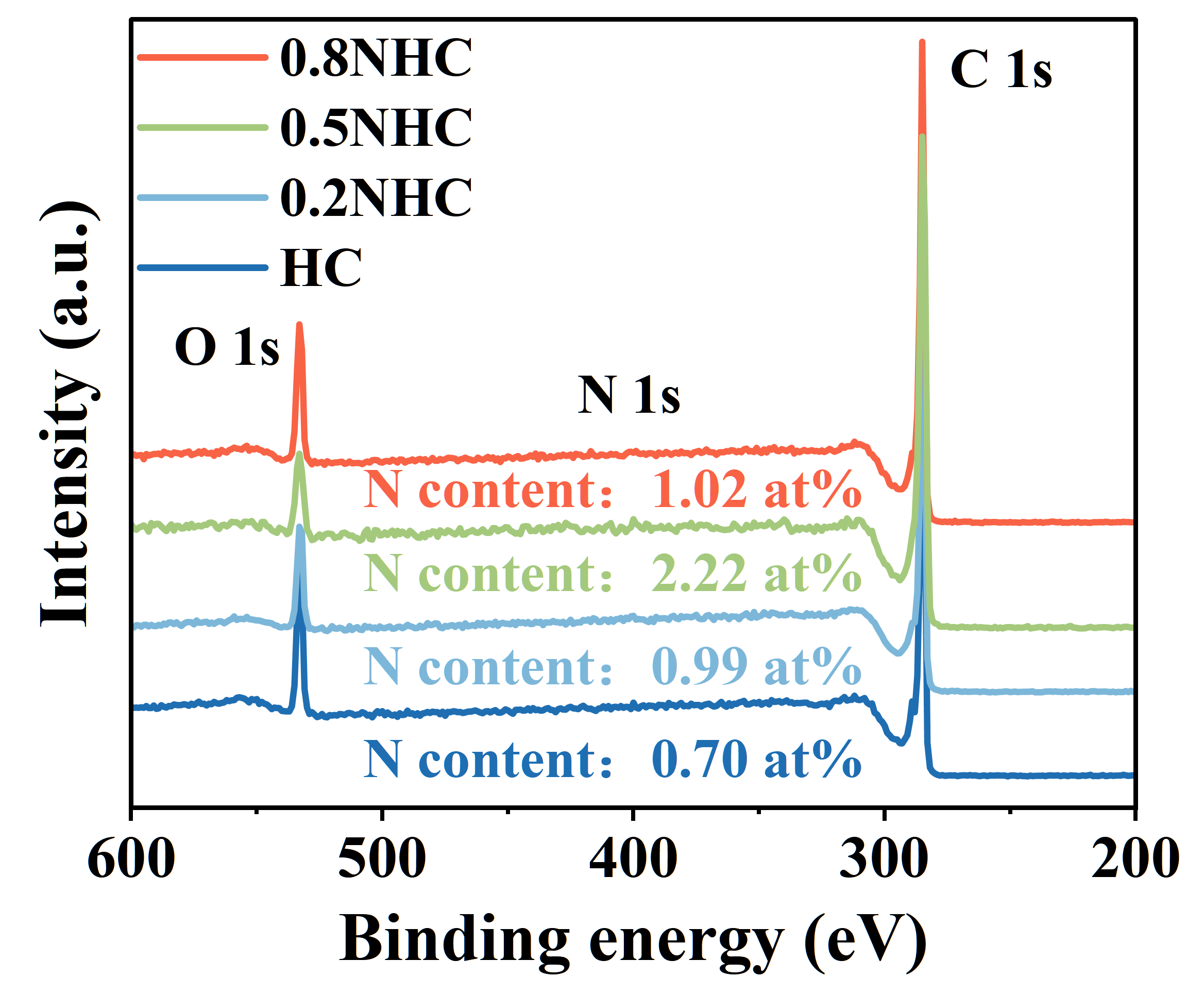


**Fig. S7** XPS survey spectra of HC and XNHC.

XPS and elemental analysis results demonstrate that the N content of 0.5NHC (2.22 wt%) is significantly higher than that of 0.8NHC (1.02 wt%), which is consistent with the EDS mapping results. This phenomenon can be attributed to the N incorporation-removal competition mechanism during high-temperature pyrolysis: an appropriate urea amount (0.5 ratio) generates active N species that sufficiently match the active sites of the carbon precursor, forming stable C-N bonds; whereas excessive urea (0.8 ratio) leads to locally high NH_3_ concentration, triggering carbon framework etching and promoting N volatilization loss in the form of HCN and NH_3_, ultimately resulting in a decreased N content.


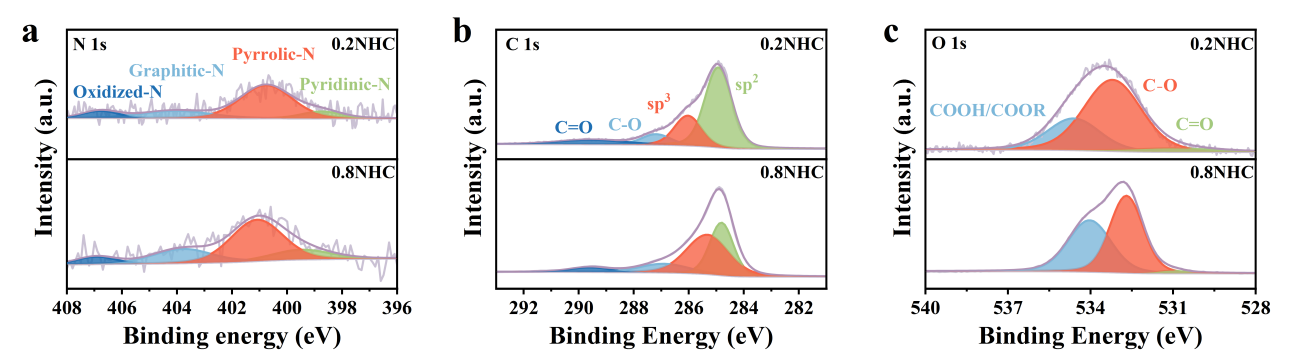


Fig. S8 a High-resolution N 1s XPS spectra of 0.2NHC and 0.8NHC. b Corresponding C 1s spectra. c Corresponding O 1s spectra.


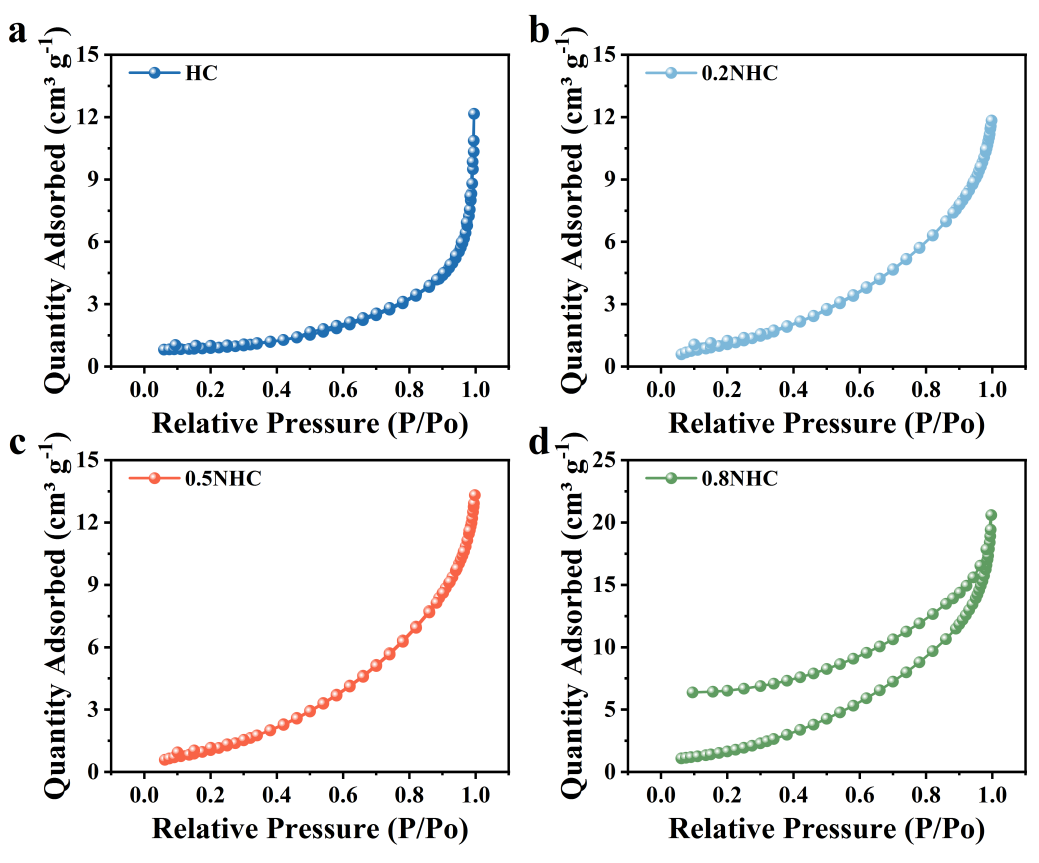


Fig. S9 Nitrogen adsorption-desorption isotherms (77K): a HC, b 0.2NHC, c 0.5NHC and d 0.8NHC.

The N_2_ adsorption-desorption isotherm of 0.8NHC (**Fig. S9d**) exhibits distinct non-closure and enhanced adsorption capacity compared to HC, 0.2NHC, and 0.5NHC, primarily attributed to excessive urea-induced structural changes. At a urea-to-HC precursor mass ratio of 0.8, urea decomposition generates abundant reactive species (e.g., NH_3_, HCN, CO_2_) that aggressively etch the carbon layers, fracturing the framework into nanofragments and transforming the short-range ordered pseudo-graphite structure into disordered cross-linked carbon layers.^[5]^ XPS and Raman analyses confirm that 0.8NHC possesses the highest sp^3^/sp^2^ ratio (1.348) and I_D_/I_G_ ratio (1.341) (**Table S1**), with abundant defects increasing the specific surface area and N_2_ adsorption sites. These high-energy sites strongly adsorb N_2_, while narrow pore constrictions lead to kinetic diffusion limitations and irreversible N_2_ trapping at -196°C—consistent with the low-pressure hysteresis (LPH) phenomenon in porous carbons.^[6]^ In contrast, moderate urea addition in other samples preserves intact frameworks, uniform pores, and moderate defect densities, enabling reversible N_2_ adsorption-desorption and closed isotherms. Notably, the over-activation of 0.8NHC increases electrode-electrolyte contact, exacerbating side reactions and irreversible sodium loss, resulting in inferior electrochemical performance.


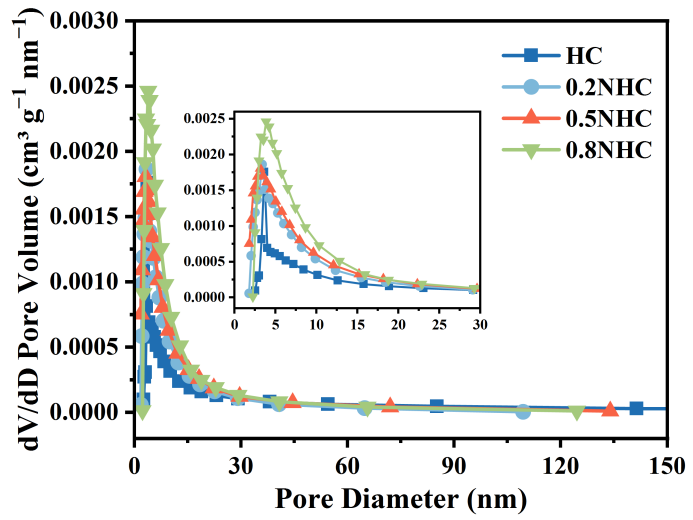


Fig. S10 The pore size distribution of HC, 0.2NHC, 0.5NHC, and 0.8NHC.


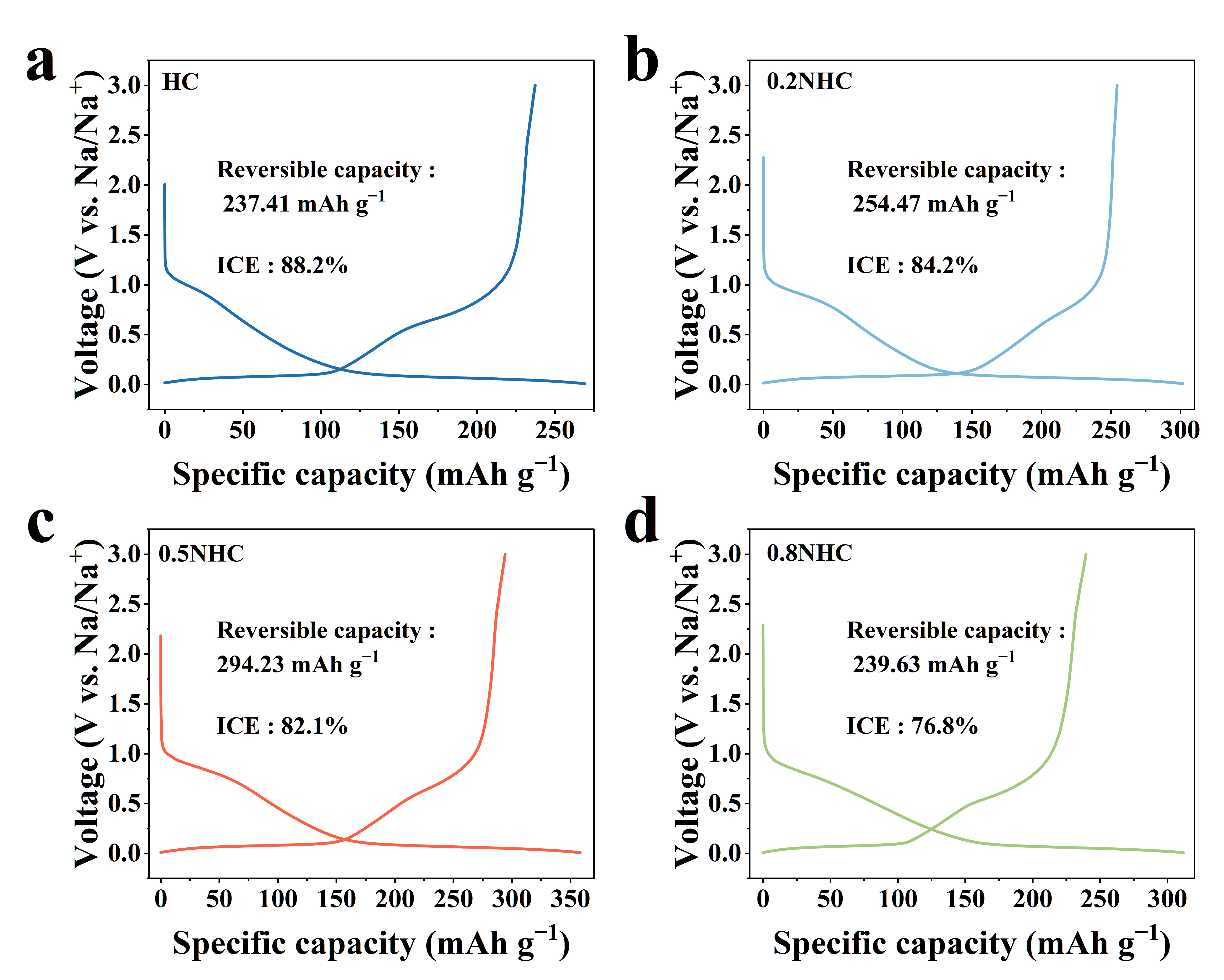


**Fig. S11** Initial GCD curves of **a** HC, **b** 0.2NHC **c** 0.5NHC, and **d** 0.8NHC.

The initial galvanostatic charge-discharge (GCD) curves of the four samples (HC, 0.2NHC, 0.5NHC, and 0.8NHC) at 0.03 A g^−1^ reveal distinct differences in sodium storage behavior, with 0.5NHC standing out as the optimal precursor for presodiation due to its balanced reversible capacity and defect characteristics.


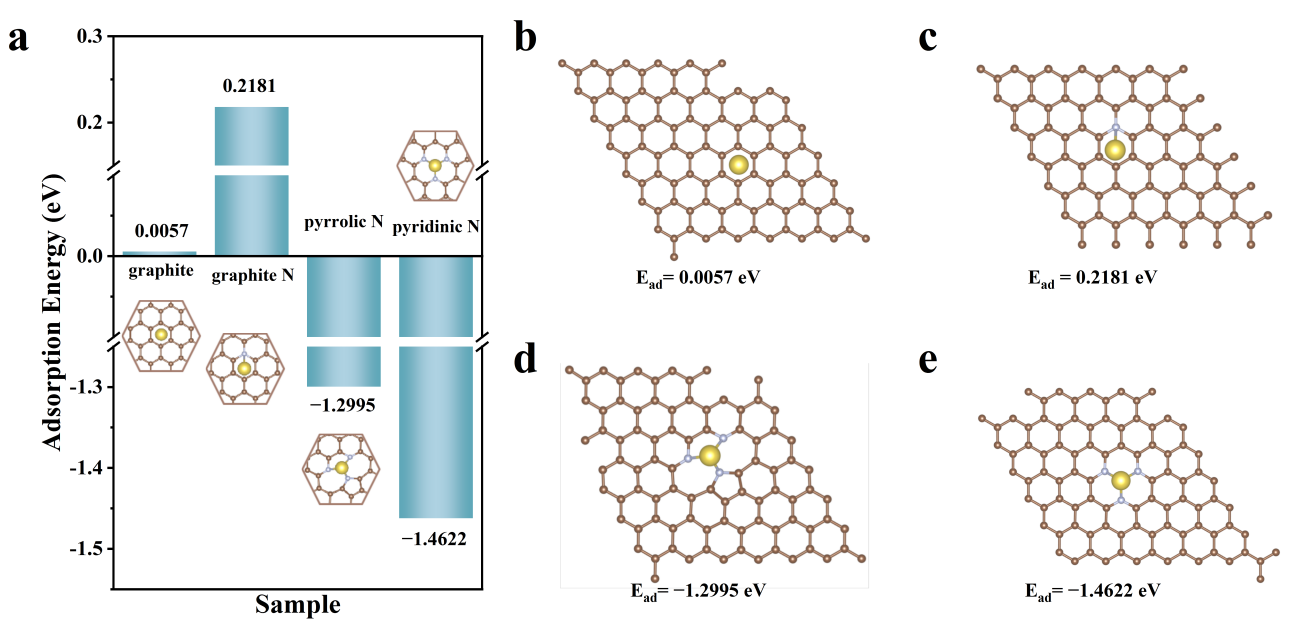


Fig. S12 a Adsorption energy of Na^+^ by different defect structures. The top-views of the sodium absorption on b graphite, c graphite-N, d pyrrolic-N and e pyridinic-N.


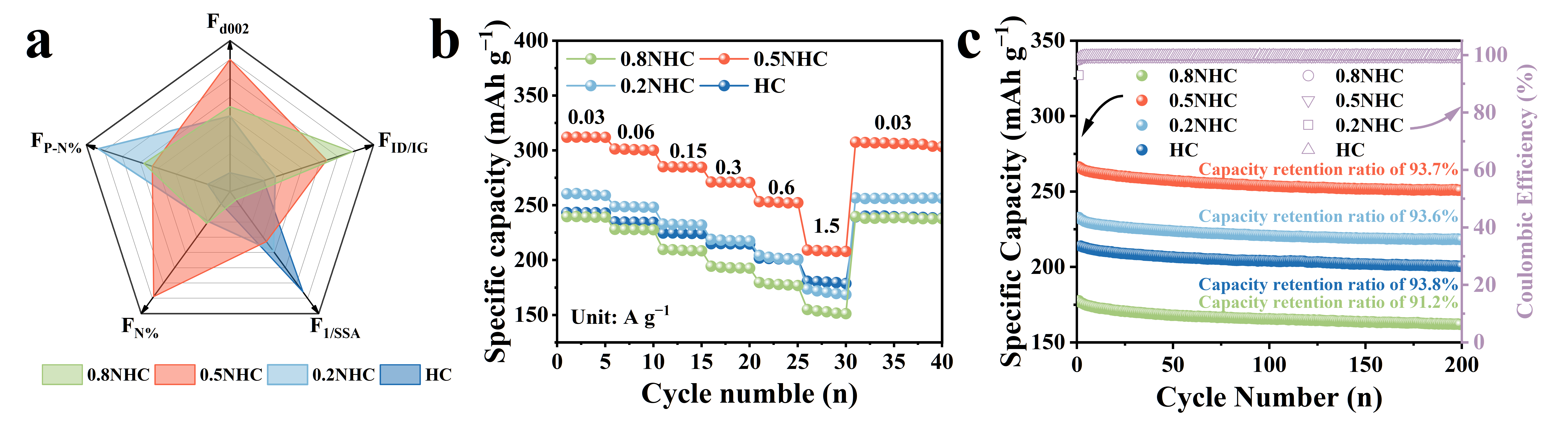


**Fig. S13 a** The influence factors of contributions to capacity (P-N%, N%, and 1/SSA are total nitrogen content, pyrrolic-N (N-5) content, and the reciprocal of specific surface area, respectively). **b** Rate performance of HC and XNHC anodes. **c** Cycling performance of HC and XNHC anodes at a current density of 0.3 A g^−1^.

0.5NHC achieves the optimal balance among expanded interlayer spacing (0.384 nm), optimized defect density (I_D_/I_G_=1.269), and structural integrity, particularly possessing the highest pyrrolic nitrogen (N-5) content that serves as reversible Na^+^ adsorption sites and enhances cation supply during presodiation. In contrast, although 0.8NHC exhibits higher defect density, excessive urea leads to over-etching of the carbon framework, excessively large specific surface area (36.511 m^2^ g^−1^), and loss of N-5 sites, aggravating side reactions and irreversible Na⁺ trapping. Electrochemical tests further confirm that 0.5NHC delivers the highest reversible capacity (294.23 mAh g^−1^), outstanding rate capability, and cycling stability. Thus, the comprehensive balance between structural characteristics and sodium storage performance makes 0.5NHC the most suitable sample for subsequent presodiation treatment.


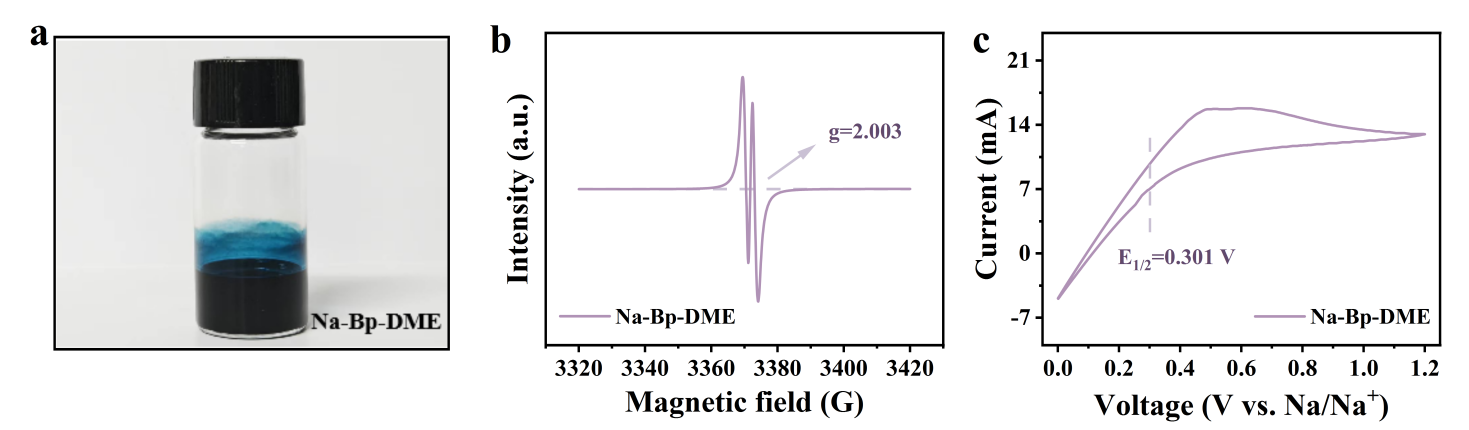


Fig. S14 a Na-Bp-DME presodiating solution. b Electron paramagnetic resonance (EPR) spectrum of Na-Bp-DME presodiating solution. c CV curve of Na-Bp-DME presodiating solution at a scan rate of 2 mV s^−1^.


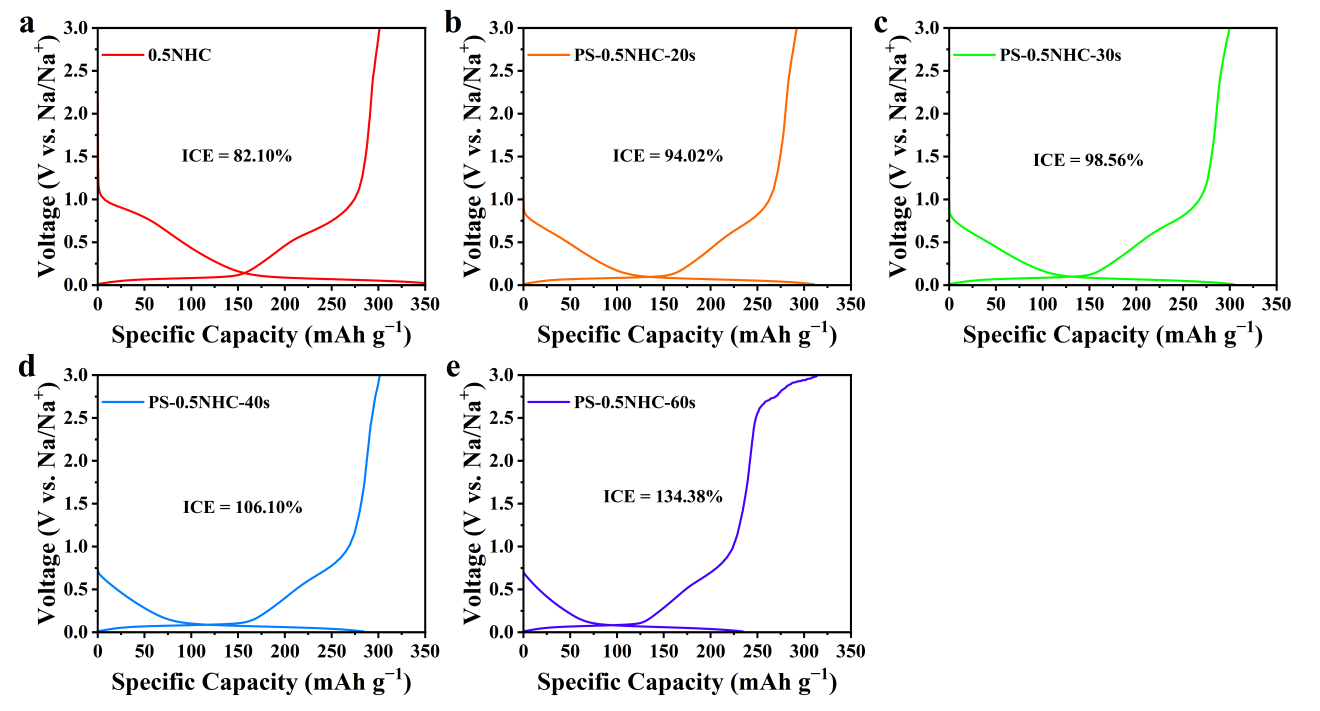


Fig. S15 Charge-discharge curves of 0.5NHC electrode at different presodiation times (0.03 A g^−1^). Presodiation time: a 0 s, b 20 s, c 30 s, d 40 s and e 60s.


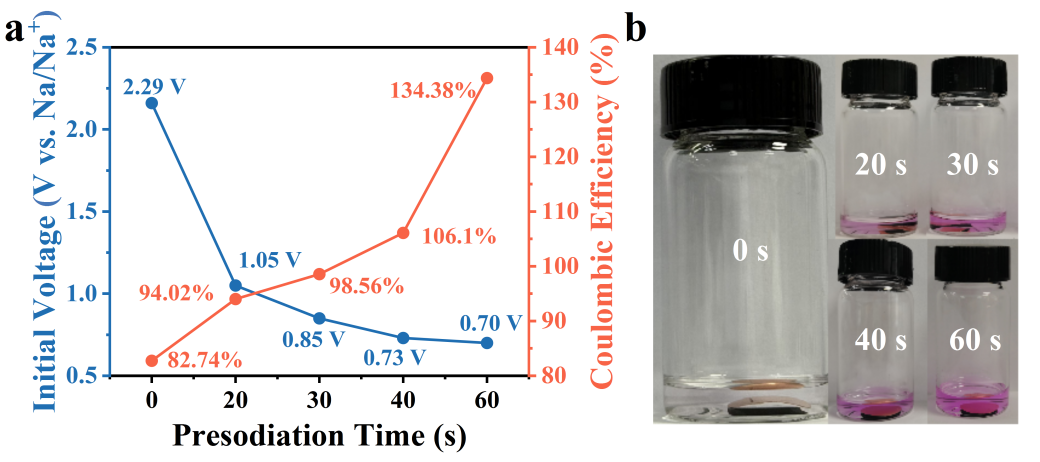


Fig. S16 Under different pre-sodiation times: a Initial Coulombic efficiency vs. open circuit voltage curve of the PS-0.5NHC anode half-cell; b Comparison diagram of color development degree of the PS-0.5NHC anode sheet in ethanol containing phenolphthalein.


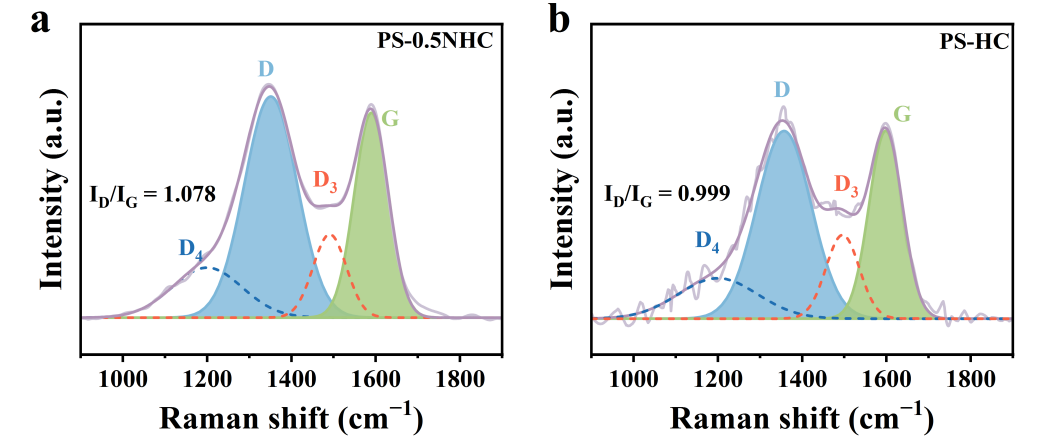


Fig. S17 Raman spectrums of a PS-0.5NHC and b PS-HC obtained with a 532 nm laser excitation.


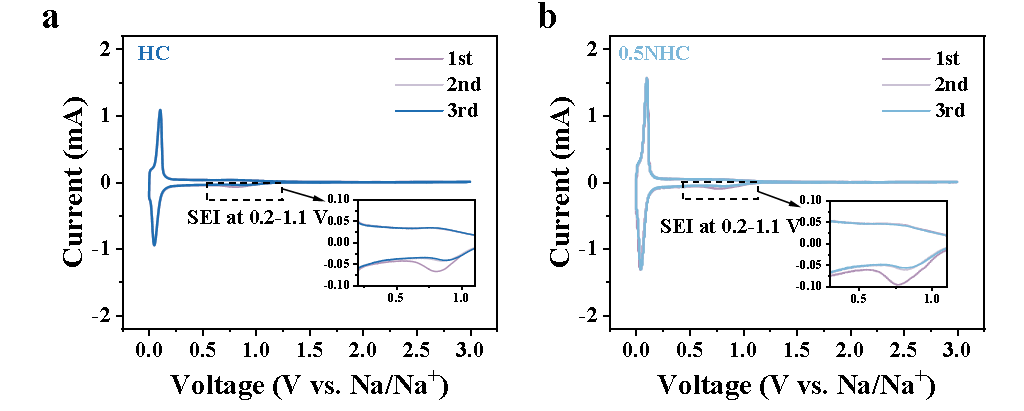


Fig. S18 CV curves of a HC and b 0.5NHC anodes in the first three cycles. (scan rate: 0.1 mV s^−1^, voltage range: 0.01–3.0 V vs. Na/Na^+^).


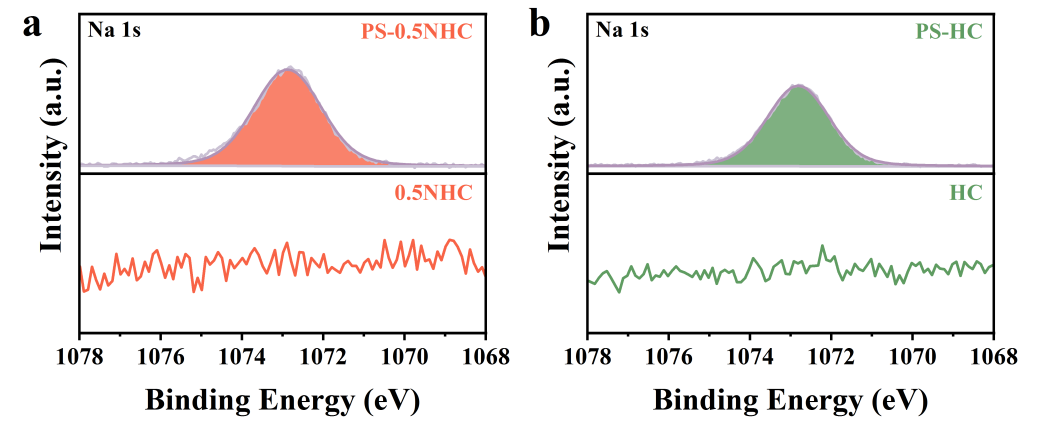


Fig. S19 N 1s XPS high-resolution spectra: a 0.5NHC and PS-0.5NHC, b HC and PS-HC.

Fig. S20 XPS survey spectra of PS-0.5NHC and PS-HC.


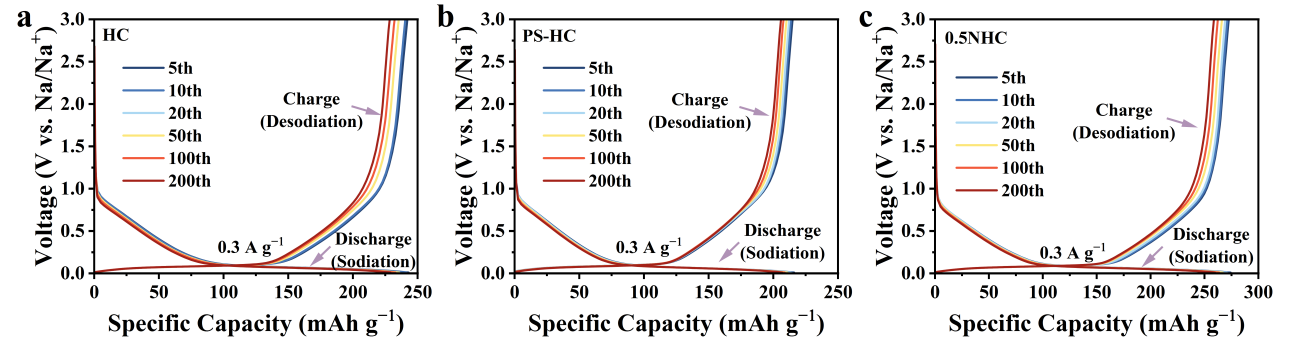


Fig. S21 Charge-discharge curves of a HC, b PS-HC and c 0.5NHC at 0.3 A g^−1^ (5th, 10th, 20th, 50th, 100th, 200th cycles).


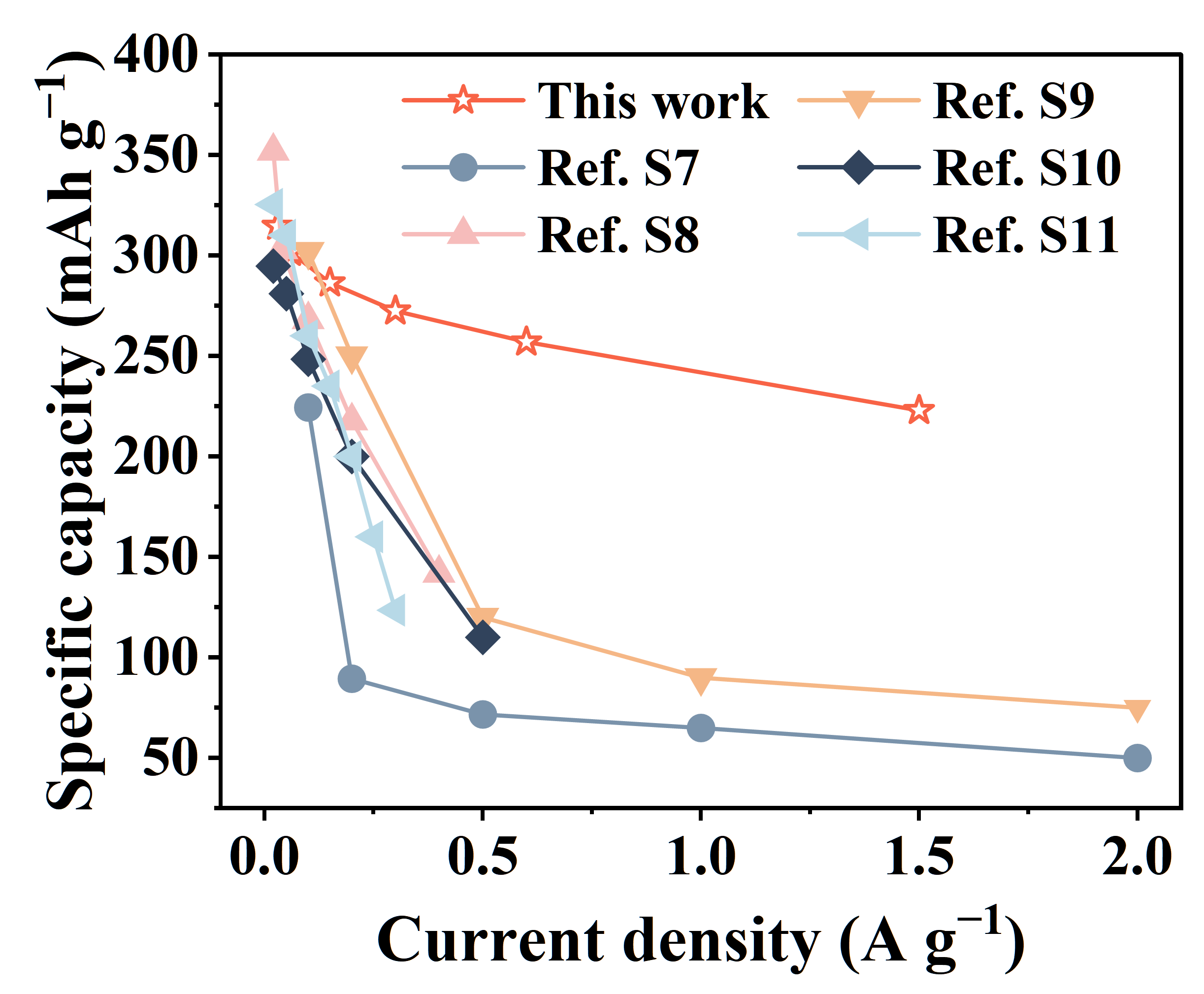


**Fig. S22** Rate capability comparison of PS-0.5NHC (this work) with previously reported coconut-shell-derived hard carbon anodes at various current densities.^[7-11]^


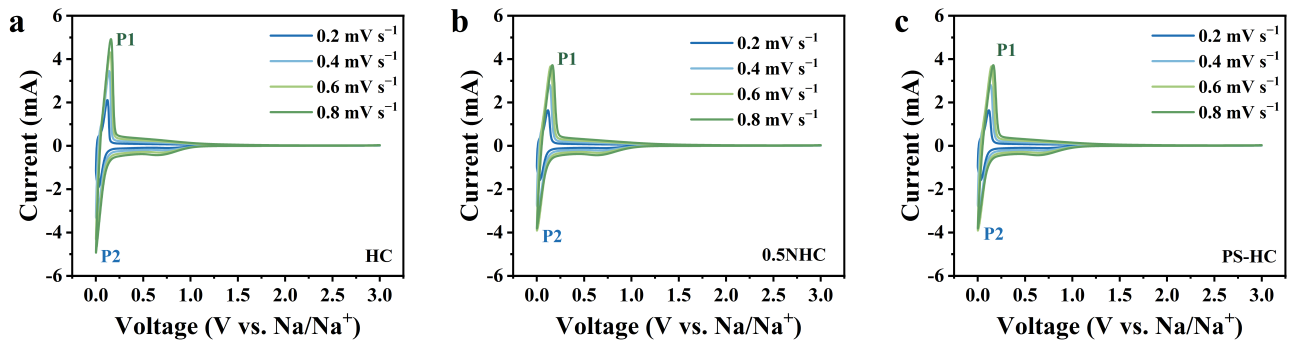


Fig. S23 CV curves of a HC, b 0.5NHC and c PS-HC anodes at different scan rates (scan rates: 0.2, 0.4, 0.6, 0.8 mV s^−1^; voltage range: 0.01–3.0 V vs. Na/Na^+^; 3nd cycle shown).


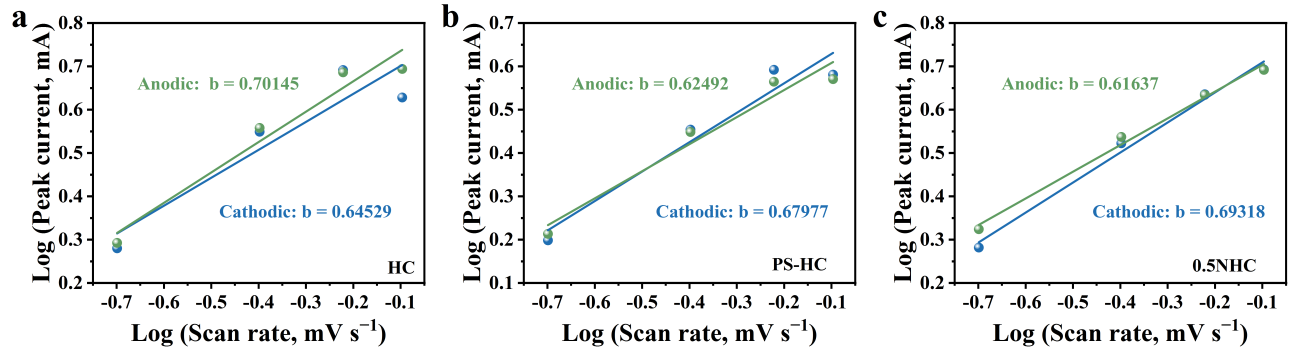


Fig. S24 Logarithmic plots of peak current vs. scan rate (log(i_P_)-log(v)) and corresponding linear fitting curves for the cathodic and anodic peaks of anode: a HC, b PS-HC and c 0.5NHC.


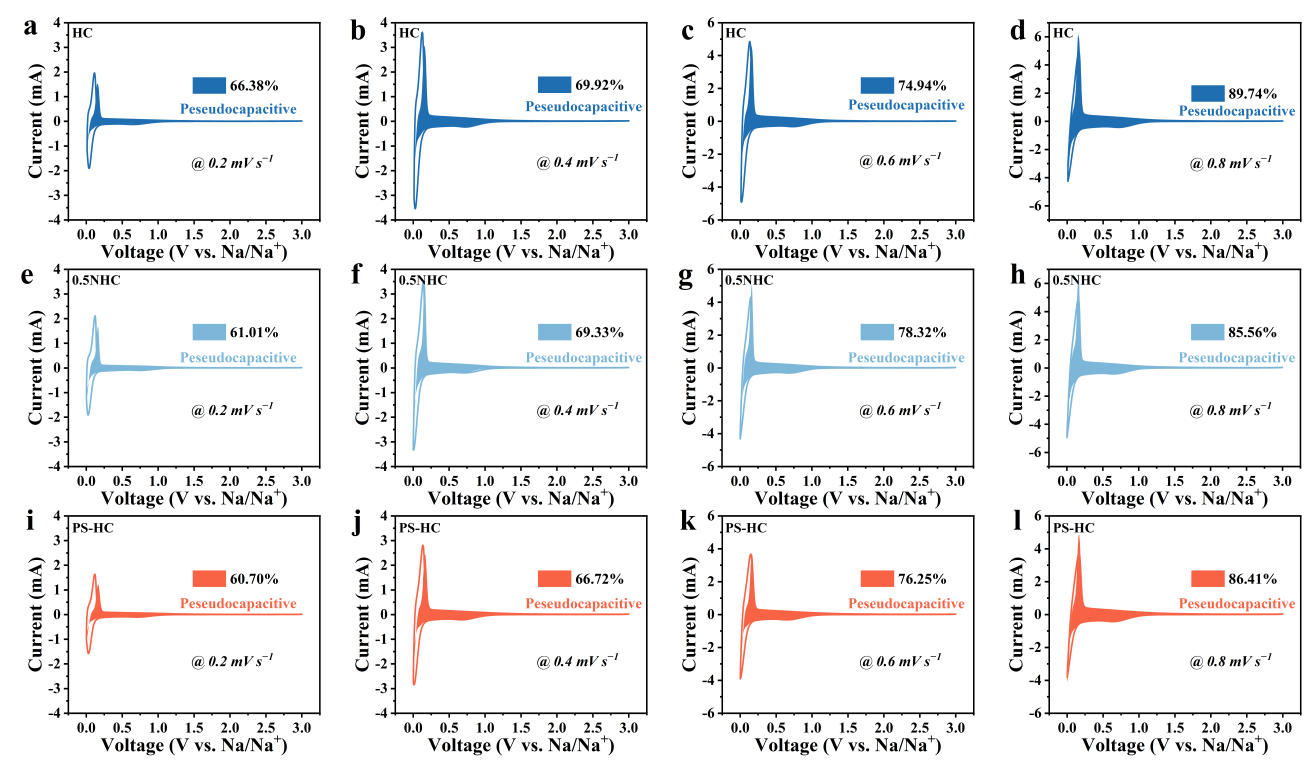


Fig. S25 Capacitive contributions of a–d HC, e–h 0.5NHC and i–l PS-HC anode materials at different scan rates. Specific scan rates (0.2, 0.4, 0.6, 0.8 mV s^−1^) are indicated in each subfigure.


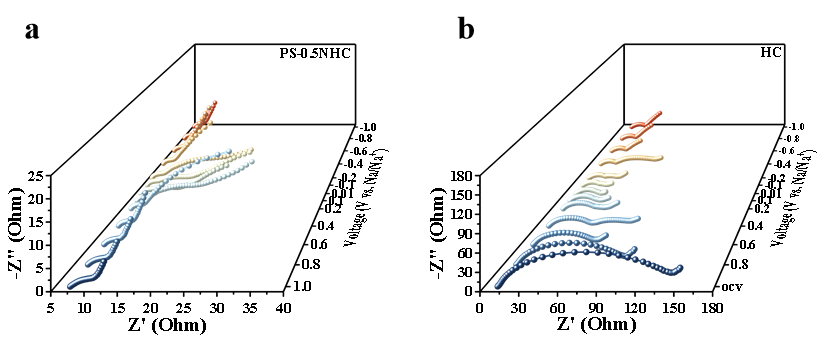


Fig. S26 Nyquist plots of a PS-0.5NHC and b HC anode obtained via in-situ electrochemical impedance spectroscopy (EIS) during GCD (0.01–3.0 V vs. Na/Na^+^).


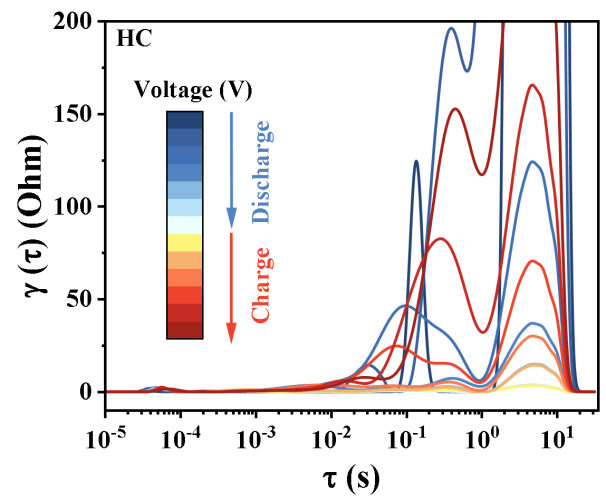


Fig. S27 Distribution of relaxation times (DRT) plots derived from in-situ EIS of the HC anode during GCD (0.01–3.0 V vs. Na/Na^+^).


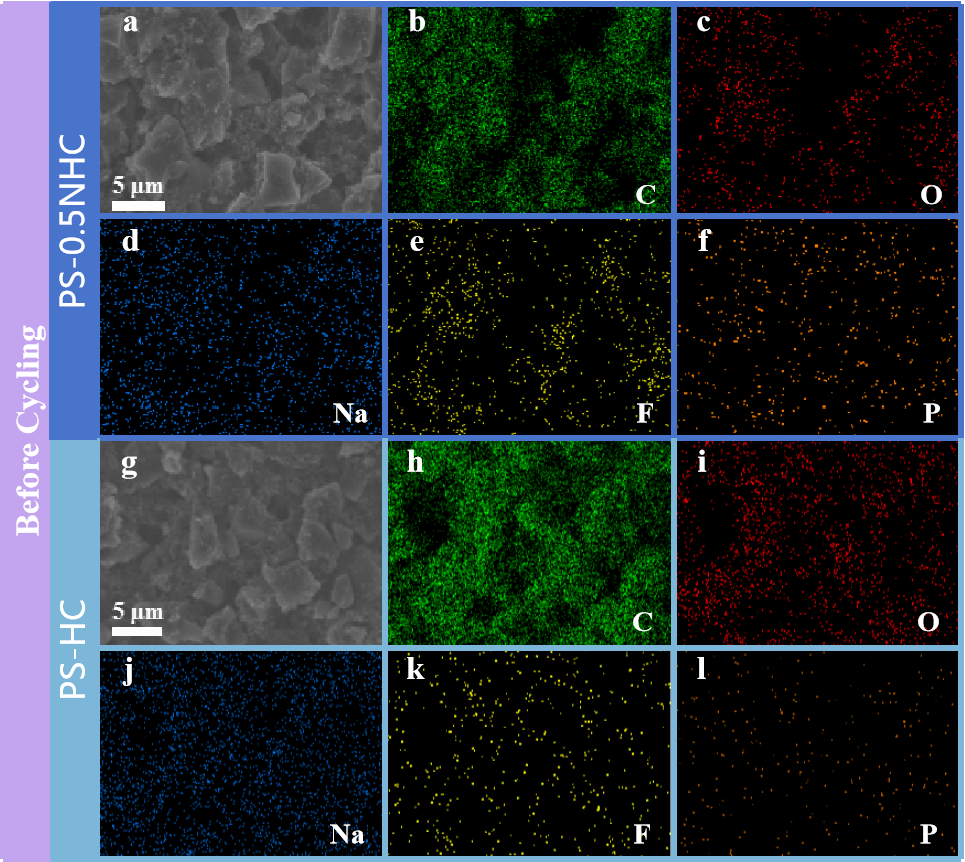


Fig. S28 SEM-EDS mapping of a-f PS-0.5NHC and g-l PS-HC anode sheets before cycling.


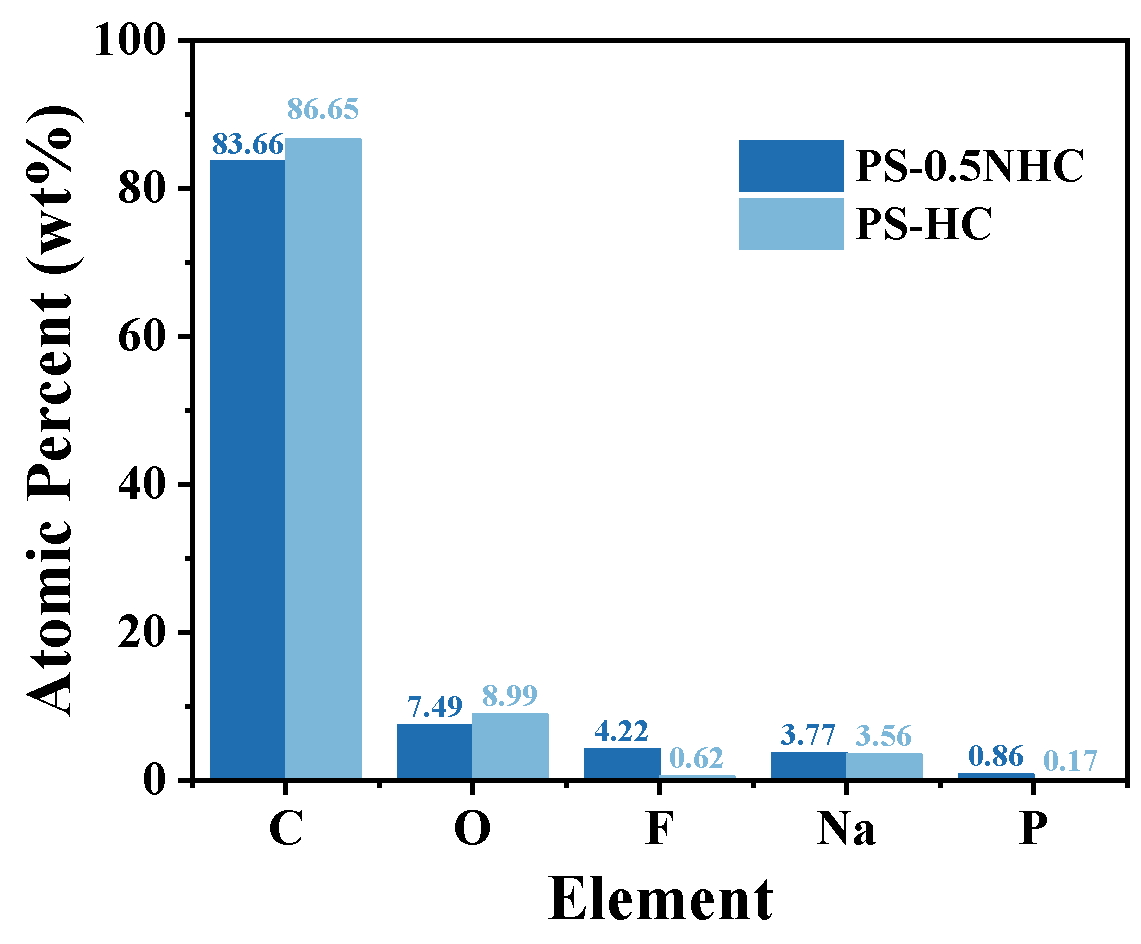


Fig. S29 The atomic percent of elements in PS-0.5NHC and PS-HC anode sheets before cycling.


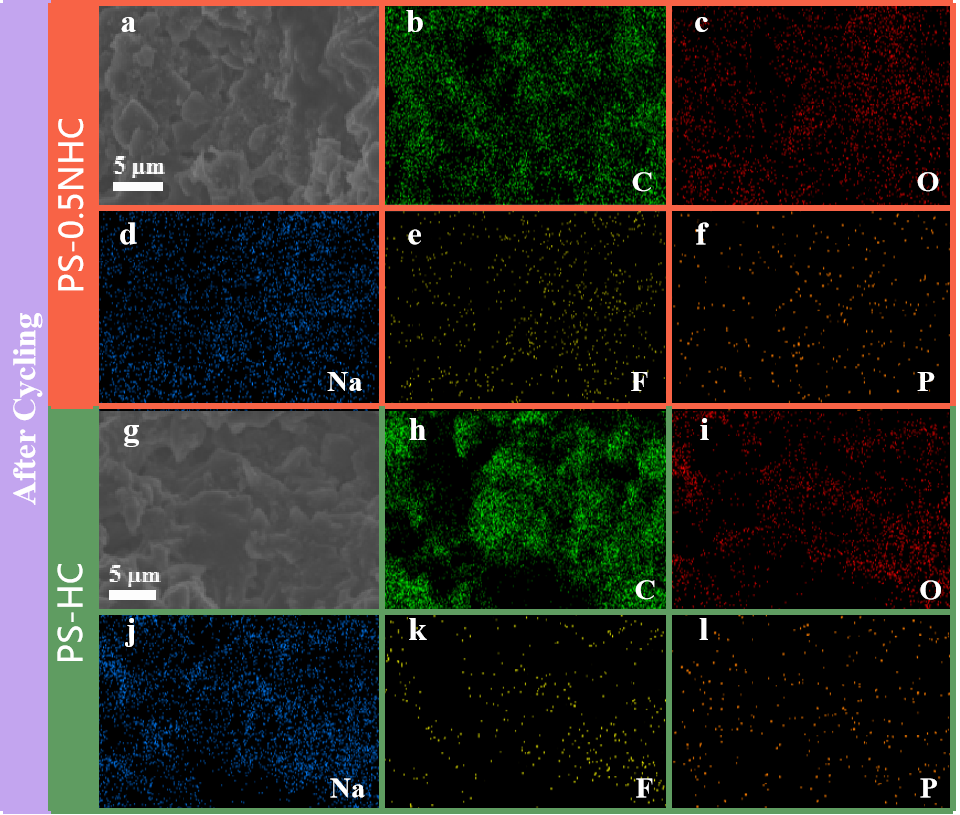


Fig. S30 SEM-EDS mapping of a-f PS-0.5NHC and g-l PS-HC anode sheets after cycling.


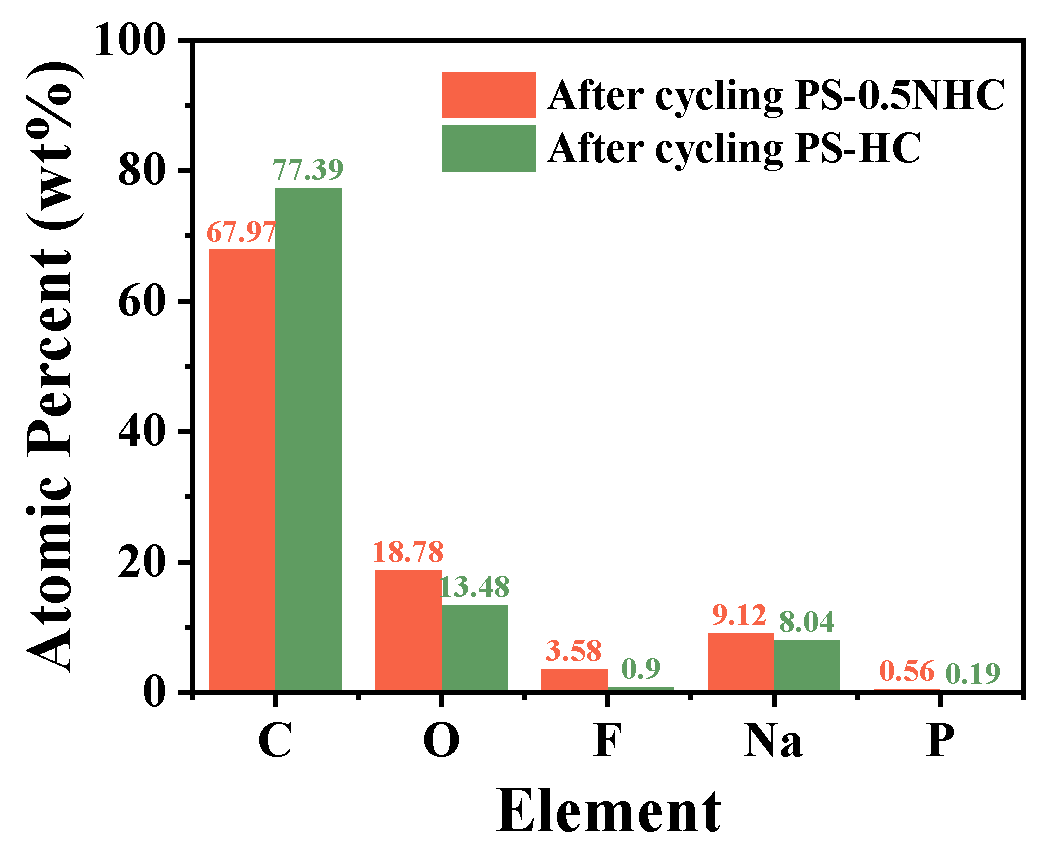


Fig. S31 The atomic percent of elements in PS-0.5NHC and PS-HC anode sheets after cycling.


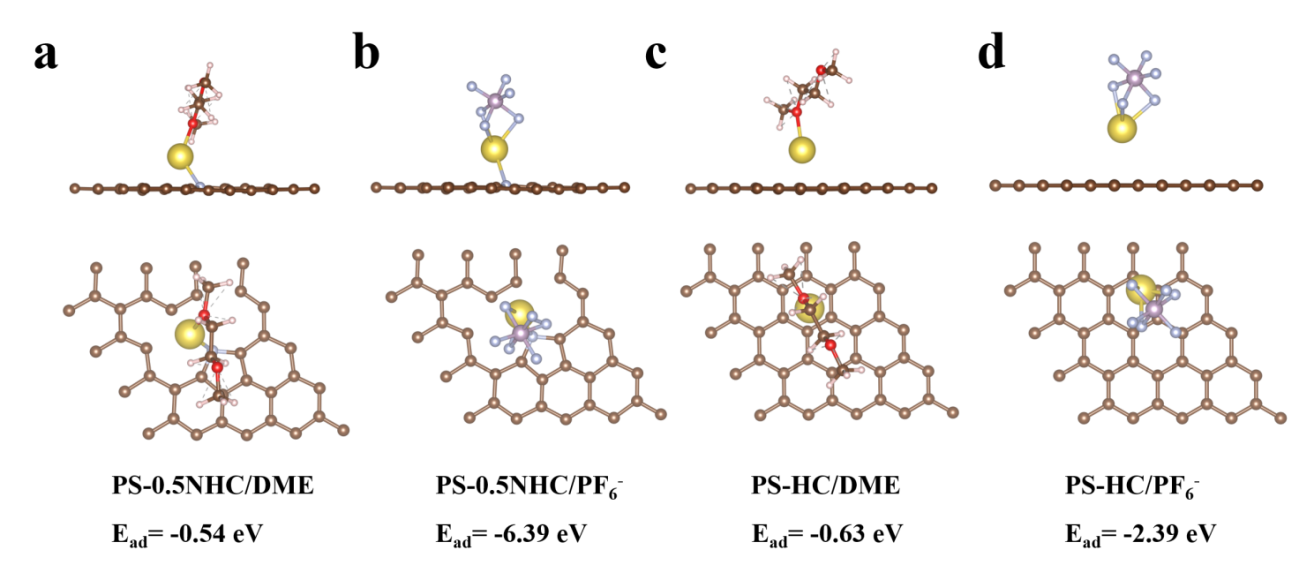


Fig. S32 Adsorption models of a PS-0.5NHC/DME, b PS-0.5NHC/PF_6_^−^, c PS-HC/DME and d PS-HC/PF_6_^−^ system (side and top views).


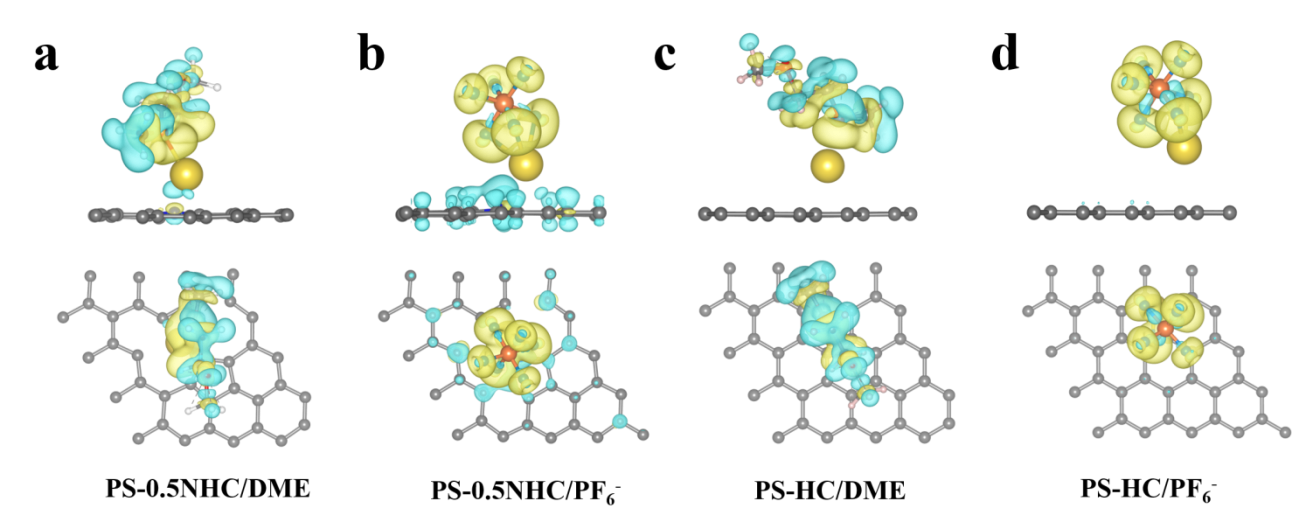


Fig. S33 Differential Charge density isosurfaces for a PS-0.5NHC/DME, b PS-0.5NHC/PF_6_^−^, c PS-HC/DME and d PS-HC/PF_6_^−^ system (side and top views).


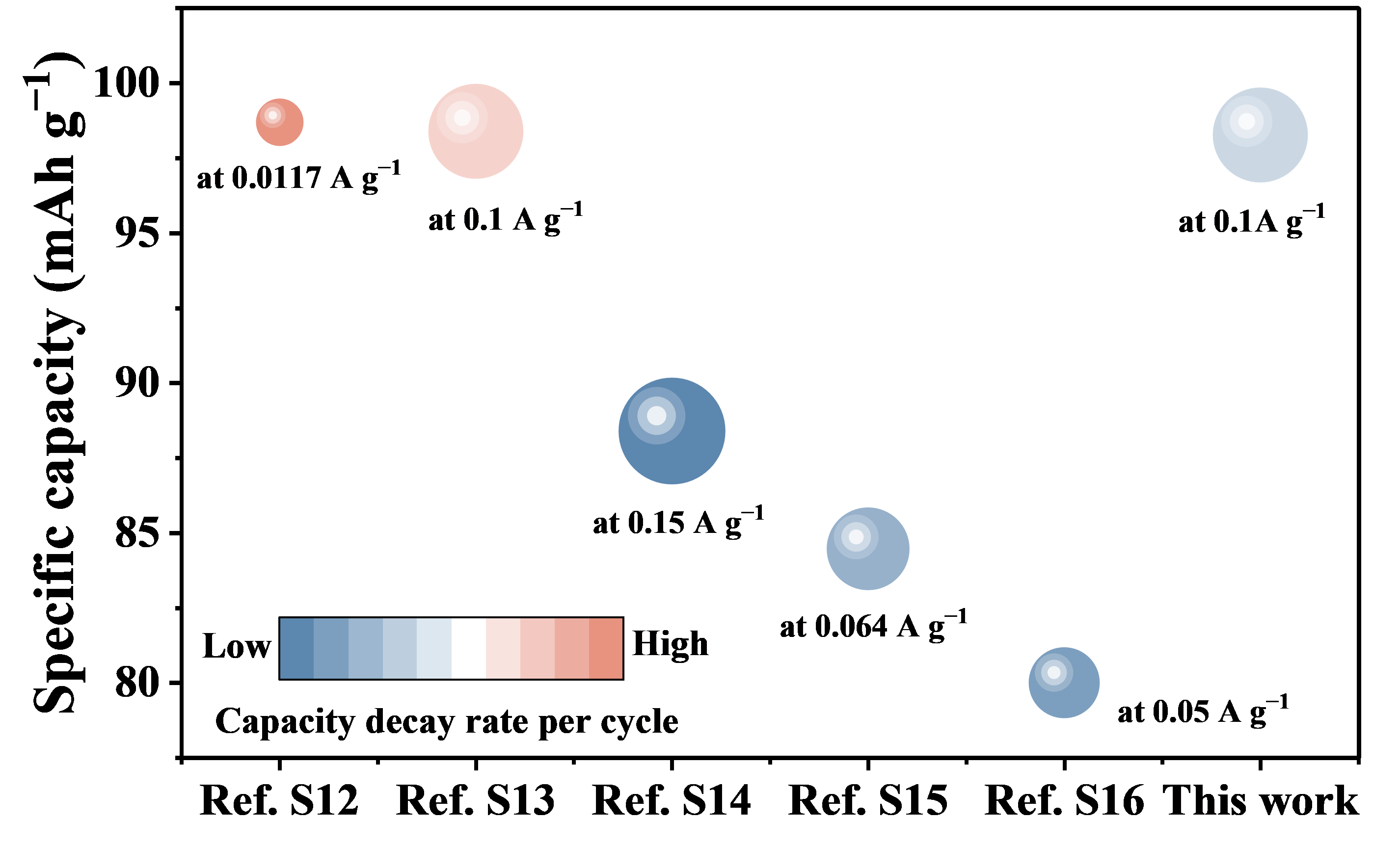


Fig. S34 Comparison of full-cell performance between PS-0.5NHC//NVP (this work) and previously reported hard carbon-based full cells..^[12-16]^

Table S1 Physical paramaters of HC, 0.2NHC, 0.5NHC and 0.8NHC carbon materials.

| Sample | d_002_  **(Å)** | R | L_a_  **(Å)** | L_c_  **(Å)** | *I*_D_/*I*_G_ | *S*_BET_  (m^2^ g^−1^) | **sp^3^/sp^2^** |
| --- | --- | --- | --- | --- | --- | --- | --- |
| HC | 3.72 | 2.81 | 30.86 | 8.79 | 1.094 | 3.035 | 0.279 |
| 0.2NHC | 3.78 | 2.39 | 27.84 | 8.65 | 1.126 | 5.110 | 0.374 |
| 0.5NHC | 3.84 | 2.16 | 26.35 | 8.44 | 1.269 | 6.097 | 0.518 |
| 0.8NHC | 3.79 | 2.02 | 25.84 | 8.38 | 1.341 | 36.511 | 1.348 |

**Table S2.** Fitting parameters of equivalent circuit elements for HC, 0.5NHC, PS-HC and PS-0.5NHC electrodes obtained from EIS analysis.

| Sample | **Rs (Ω)** | Rct (Ω) | **CPE (S·s^n^)** | **Wo** | χ^2^ (×10^-4^) |
| --- | --- | --- | --- | --- | --- |
| HC | 10.49 | 242.5 | 5.2×10^-6^ | 3.14 | 5.94 |
| 0.5NHC | 5.608 | 42.02 | 5.5×10^-6^ | 33.81 | 0.80 |
| PS-HC | 15.09 | 64.58 | 6.1×10^-6^ | 1.97 | 1.34 |
| PS-0.5NHC | 6.484 | 12.47 | 4.8×10^-6^ | 13.1 | 0.45 |

All EIS data were fitted using ZView software, and the fitting parameters with their associated errors are summarized in **Table S2**. The chi-squared (χ²) values for all fits are on the order of 10^−4^, indicating excellent fitting quality and good agreement between the experimental data and the fitted model.

**Table S3.** Comparison of electrochemical performance of coconut-shell-derived hard carbon anodes.

| Reference | **ICE (%)** | **Reversible Capacity**  **(mAh g⁻¹)** | Rate Capability | | Cycling Stability |
| --- | --- | --- | --- | --- | --- |
| This Work (PS-0.5NHC) | 98.54 (half-cell) 91.53 (full-cell) | ~301 @0.03 A g^−1^ | | ~222 @1.5 A g^−1^ | 90.6% after 1000 cycles @1.5 A g^−1^ |
| [7] | 78.2 (HC-1300) | ~221 @0.1 A g^−1^ | | 54.5 @2 A g^−1^ (HC-1200) | 224.36 mAh g^−1^ after 100 cycles @0.1 A g^−1^ |
| [8] | Not reported | ~320 @0.02 A g^−1^ | | 142.0 @0.4 A g^−1^ | 88.1% after 50 cycles @0.02 A g^−1^ |
| [9] | 69.8 (CHC-3) | 292.6 @0.1 A g^−1^ | | ~150 @1.0 A g^−1^ | 94% after 100 cycles @0.1 A g^−1^ |
| [10] | 62.36 (e-HC-10) | 297.07 @0.02 A g^−1^ | | ~67 @1.0 A g^−1^ | ~75% after 500 cycles @0.1 A g^−1^ |
| [11] | 93.2-95.0 | 326.3-335.6 @0.02 A g^−1^ | | 122.9 @0.3 A g^−1^ | 92.7% after 200 cycles @0.02 A g^−1^ |

**References**

[1] M. Liu, J. Zhang, S. Guo, et al., "Chemically Presodiated Hard Carbon Anodes with Enhanced Initial Coulombic Efficiencies for High-Energy Sodium Ion Batteries," *ACS Applied Materials & Interfaces* 12 (2020):17620–17627, <https://doi.org/10.1021/acsami.0c02230>.

[2] P. Hohenberg, W. Kohn, "Inhomogeneous Electron Gas," *Physical Review* 136 (1964):B864–B871, <https://doi.org/10.1103/PhysRev.136.B864>.

[3] W. Kohn, L. J. Sham, "Self-Consistent Equations Including Exchange and Correlation Effects," *Physical Review* 140 (1965):A1133–A1138, <https://doi.org/10.1103/PhysRev.140.A1133>.

[4] J. P. Perdew, K. Burke, M. Ernzerhof, "Generalized gradient approximation made simple," *Physical Review Letters* 77 (1996):3865,

[5] Y. Liu, S. Dai, J. Deng, et al., "Precise control of the resin-based hard carbon pseudo graphite and closed pores structure to enhance sodium storage capacity," *Journal of Colloid and Interface Science* 686 (2025):136–150, <https://doi.org/10.1016/j.jcis.2025.01.168>.

[6] T. Zelenka, L. Zelená, C. Abreu‐Jaureguí, et al., "On the Low‐Pressure Hysteresis (LPH) in Gas Sorption Isotherms of Porous Carbons," *Small* (2024):<https://doi.org/10.1002/smll.202311990>.

[7] Y. Cheng, J. Zhao, L. Zhang, et al., "The effect of thermal treatment temperature on the crystal structure and electrochemical performance of the coconut shell-based hard carbon," *Solid State Ionics* 402 (2023):116374, <https://doi.org/10.1016/j.ssi.2023.116374>.

[8] H. Xiao, F. Wang, J. Peng, et al., "Zinc-assisted modification of hard carbon for enhanced sodium-ion storage," *Journal of Electroanalytical Chemistry* 978 (2025):118889, <https://doi.org/10.1016/j.jelechem.2024.118889>.

[9] G. Zhao, X. Deng, Z. Wang, X. Wang, "Boosting sodium storage performance of hard carbons by regulating surface oxygen functionalities," *Materials Letters* 396 (2025):138783, <https://doi.org/10.1016/j.matlet.2025.138783>.

[10] Q. Wang, L. Du, S. Wang, et al., "Biomass-Derived Hard Carbon Anodes Processed with Deep Eutectic Solvents for High-Performance Sodium-Ion Batteries," *ACS Omega* 10 (2025):23620–23628, <https://doi.org/10.1021/acsomega.5c02496>.

[11] M. Liu, L. Xin, M. Ning, Y. Liu, "Dual-Topology-Driven Oxygenated Lattice Memory and Carbon-to-Oxygen Substitution Enable Graphite-like Subcrystalline Carbon for Na-Ion Batteries," *ACS Nano* 19 (2025):34248–34263, <https://doi.org/10.1021/acsnano.5c11196>.

[12] S. Xiao, H. Li, Z. Huang, et al., "Engineering of Closed Pores and Transport Channels in Polymer-Derived Hard Carbon via Chemical Vapor Deposition for Sodium-Ion Batteries," *ACS Nano* 20 (2026):11096–11107, <https://doi.org/10.1021/acsnano.5c21256>.

[13] W. He, W. He, S. Zheng, et al., "Atomic-level Fe/N-coordinated biomass hard carbon for fast-charging sodium-ion batteries," *Energy Storage Materials* 88 (2026):105055, <https://doi.org/10.1016/j.ensm.2026.105055>.

[14] S. Lin, H. Zhang, Z. Huang, et al., "Dual-functional chemical pre-sodiation of carbon-coated hard carbon anodes with initial Coulombic efficiency up to 99.5% for sodium-ion batteries," *Science China Materials* (2026):<https://doi.org/10.1007/s40843-025-3944-9>.

[15] S. Mahato, A. Das, K. Biswas, "Experimental and theoretical investigation on boron, phosphorus dual doped hard carbon as anode for sodium-ion battery," *Journal of Energy Storage* 104 (2024):114422, <https://doi.org/10.1016/j.est.2024.114422>.

[16] Y. Wu, Y. Mo, W. Zhou, et al., "Bifunctional crosslinking-induced structural engineering towards improved sodium storage in pitch-derived hard carbon," *Journal of Energy Chemistry* 116 (2026):134–143, <https://doi.org/10.1016/j.jechem.2025.12.048>.
